# A comprehensive mouse kidney atlas enables rare cell population characterization and robust marker discovery

**DOI:** 10.1016/j.isci.2023.106877

**Published:** 2023-05-18

**Authors:** Claudio Novella-Rausell, Magda Grudniewska, Dorien J.M. Peters, Ahmed Mahfouz

**Affiliations:** 1 Department of Human Genetics, Leiden University Medical Centre, 2333 ZA Leiden, the Netherlands; 2 GenomeScan, 2333 BZ Leiden, the Netherlands; 3 Leiden Computational Biology Center, Leiden University Medical Center, Leiden, the Netherlands; 4 Delft Bioinformatics Lab, Delft University of Technology, Delft, the Netherlands

**Keywords:** Biological sciences, Cell biology, Transcriptomics

## Abstract

The kidney’s cellular diversity is on par with its physiological intricacy; yet identifying cell populations and their markers remains challenging. Here, we created a comprehensive atlas of the healthy adult mouse kidney (MKA: Mouse Kidney Atlas) by integrating 140.000 cells and nuclei from 59 publicly available single-cell and single-nuclei RNA-sequencing datasets from eight independent studies. To harmonize annotations across datasets, we built a hierarchical model of the cell populations. Our model allows the incorporation of novel cell populations and the refinement of known profiles as more datasets become available. Using MKA and the learned model of cellular hierarchies, we predicted previously missing cell annotations from several studies. The MKA allowed us to identify reproducible markers across studies for poorly understood cell types and transitional states, which we verified using existing data from micro-dissected samples and spatial transcriptomics.

## Highlights

• MKA integrates 59 publicly available datasets of the healthy mouse kidney
• Comprehensive harmonization of annotations and classification of missing labels
• Robust marker identification for known and previously overlooked cell types
• Cell type classification using MKA outperforms any individual dataset

## Graphical abstract

**Figure:**
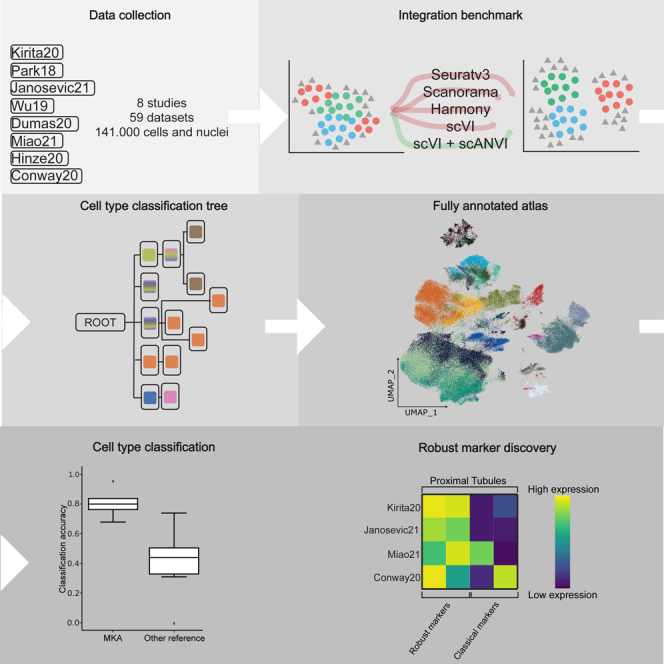

## Introduction

Kidneys are organs with a high degree of cellular complexity reflected in an array of different renal functions: from filtering the blood, regulating water homeostasis, production of hormones, to excretion of waste products. These diverse functions are driven by distinct anatomical structures called nephrons. Each nephron comprises more than tens of highly specialized cell types, including abundant epithelial cells supported by vascular, stromal, and immune cells.^1^ Notably, the function and nomenclature of cells that assemble the nephron depend on their location relative to the main tubular structures: the proximal tubule, loop of Henle, distal convoluted tubules, and the collecting duct.^2^

**Figure 1:**
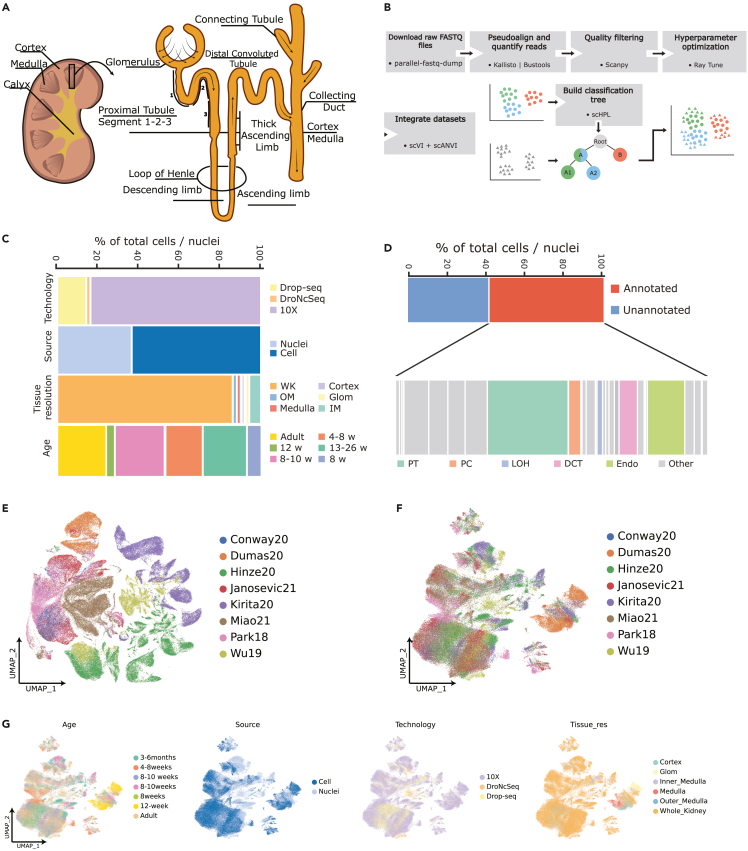
Generation of the mouse kidney atlas from eight independent studies (A) Schematic of a kidney and a nephron. Arrows indicate the flux of glomerular filtrate through the tubular segments. (B) Workflow guiding the generation of the mouse kidney atlas. Colors represent different hypothetical cell type annotations from two independent studies (A, B, and A1, A2), whereas shapes depict originally annotated (circle) or unannotated (triangles) cells and/or nuclei. (C) Metadata information across all datasets. Age of the animals is represented in weeks or months (w, m) when available, otherwise an overall age estimator is provided (Adult). Tissue resolution varied from whole kidney (WK), cortex, medulla to more selected regions, such as Outer Medulla (OM), Inner Medulla (IM), or Glomerulus (Glom). The suspension type was either single cell or single nuclei sequenced using Drop-seq, DroNc-Seq or 10x Genomics. (D) Proportion of all annotated cell types across all datasets. Relevant cell types in the nephron are highlighted, namely Proximal Tubule cells (*PT*), Principal Cells (*PC*), Loop of Henle cells (*LOH*), Distal Convoluted Tubule cells (*DCT*) and Endothelial cells (*Endo*). (E) Uni-form Manifold Approximation and Projection (UMAP) embedding of all used datasets prior integration. Colors correspond to the different datasets. (F) UMAP visualization of merged datasets following integration and batch correction (see STAR Methods). sCell: single-cell, sNuc: single-nuclei. (G) UMAP representations of the 140K cells and nuclei after integration. Relevant metadata was extracted for each of the datasets. Age of the animals is represented in weeks or months when available, otherwise an overall age estimator is provided (Adult).

More than 150 L of filtrate are reabsorbed by the nephrons in a day. Most of this reabsorption occurs in the proximal tubules, which are primarily located in the cortex, the outermost portion of the kidney. Sodium gradient, generated by the activity of numerous Na⁺/K⁺-ATPase channels, drives the transport of salts, water, glucose, and amino acids, back to the bloodstream. This process requires large amounts of energy, supplied by the abundant mitochondria. Proximal tubules are thus, the most metabolically active structures in the nephron.^3^^,^^4^^,^^5^ The filtrate then enters the loop of Henle that connects the proximal and distal tubule and is most notably involved in extracellular fluid volume and blood pressure regulation, as well as Ca^2+^, Mg^2+^, and acid-base homeostasis. Through the activation of several processes required to generate a gradient of increasing osmolality from cortex to medulla, this segment also contributes to urine concentration.^6^ Finally, the filtrate travels through the distal convoluted tubule and collecting duct system, where water is reabsorbed and urine is concentrated^7^ (Figure 1A).

Although, outstanding efforts to characterize the transcriptomic profiles of the different cell types present in the kidney have been supported by the recent advances in single-cell technologies,^8^^,^^9^^,^^10^^,^^11^^,^^12^^,^^13^^,^^14^^,^^15^ the identification of markers for distinct and, in particular, rare cell types remains elusive. A substantial portion of the transcriptomic data comes from the proximal tubule and loop of Henle cells, which are one of the largest structures of the nephron.^16^ Rare cell populations usually remain undetected and the cost to profile these cells often precludes the studies of less abundant cell populations.^17^^,^^18^ The aforementioned challenges could be addressed by creating a reference atlas of the kidney that leverages the vast collection of available cells and nuclei profiled, which can then be used to annotate specific kidney cell types in a supervised manner. Integrating different datasets into a common space can overcome batch effects which occur due to the differences in library preparation protocols and data processing steps. However, in most studies to date, the annotation of cell populations is performed in an unsupervised manner, a process that is time-consuming and involves several refinement iterations.^19^ The subjective nature of this approach limits the ability to compare populations across studies due to annotation inconsistencies. Ideally, the reference atlas would account for the different resolutions at which cell populations have been annotated and include a harmonized annotation that allows for a better characterization of rare cell populations.

Here, we create an atlas of the adult healthy mouse kidney (MKA: Mouse Kidney Atlas) by integrating and harmonizing annotations from publicly available single-cell and single-nuclei transcriptomic studies. We integrated ∼140.000 cells and nuclei from 59 healthy samples sequenced in eight different studies^8^^,^^9^^,^^10^^,^^11^^,^^12^^,^^13^^,^^14^ to generate an atlas that reflects the biological component of the different samples, while accounting for technical differences. We built a hierarchical model of the cell populations present in the healthy mouse kidney that accurately predicts cell annotations in unlabeled datasets. In addition, MKA allows further integration of new datasets as they become available by relying on a progressive learning approach^20^ (Figure 1B). We show and verify novel and robust markers for both known cell types and previously unexplored rare cell populations.

## Results

### Integrated atlas accounts for technical differences among seven independent studies

To create a comprehensive atlas of the healthy mouse kidney, we downloaded the raw sequencing data (FASTQ) from eight different studies including a total of 59 samples^8^^,^^9^^,^^10^^,^^11^^,^^12^^,^^13^^,^^14^^,^^15^ (Table 1). To reduce variability in alignment rates between different genetic make-ups, we only included healthy samples with a C57BL/6 background. The raw reads of all samples were processed using the same pipeline and we recovered 140,000 cells and nuclei after filtering low quality cells and nuclei (see STAR Methods section for details). The samples included in this study differ in single-cell technology, source of material, tissue resolutions, and age of sacrifice (Figure 1C). Approximately 40% of the cells and nuclei included in this study were missing computer-readable annotations (Figure 1D). These differences can be visualized in the uni-form manifold approximation and projection (UMAP) of the data (Figure 1E), where source-specific populations were identified. To resolve these batch effects, we evaluated the performance of five batch correction methods (Seurat, Harmony, Scanorama, scVI and scVI-scANVI^21^^,^^22^^,^^23^^,^^24^^,^^25^) using their respective default parameters (Figure S1A and Table S1). The best performing method was scVI (overall score of 0.72). Notably, while algorithms, such as Seurat efficiently correct the batch effects (batch effect removal score of 0.84) compared to scVI (batch effect removal score of 0.71), the latter better maintains the biology of each individual dataset after integration (biological conservation scores of 0.72 and 0.38 for scVI and Seurat, respectively). We also observed that methods, such as Seurat overcorrected for batch differences by aligning all datasets to a common latent space. This is especially evident in the case of Dumas20, a dataset that only contains endothelial cells. Its cells are overcorrected by Seurat and hence aligned with all other datasets and cell types (Figures S1B and S1C).

**Table 1:** Dataset metadata

Abbreviation	Technology	GEO	Reference	Samples	Cell sorting	SC/SN	Dissociation	Genes per cell or nuclei	Counts per cell or nuclei	Reads (Mill.)	% of pseudoaligned reads	Available annotations
Wu18	Dropseq, 10X, DroNcSeq	GSE119531	11	4	WT	SN, SC	Mechanical	805	1482	55	76	Yes
Miao21	10X	GSE157079	14	4	WT	SC	Enzymatic	1175	2822	152	75	Yes
Park18	10X	GSE107585	8	35	WT	SC	Enzymatic	657	1374	745	76	Yes
Kirita20	10X	GSE139107	9	4	WT	SN	Mechanical	1032	1862	1481	85	Yes
Dumas20	10X	E-MTAB-8145	13	3	C, G, M	SC	Enzymatic	1229	2072	462	59	No^a^
Conway20	10X	GSE140023	12	1	WT	SC	Enzymatic + Mechanical	842	2027	343	83	No
Hinze21	Drop-seq, 10X	GSE145690	10	7	IM, OM, C, WT	SC	Mechanical	711	1419	177	80	No
Janosevic21	10X	GSE151658	15	1	WT	SC	Enzymatic + Mechanical	1608	4100	407	77	No^b^

10X: 10X Genomics, SC: single-cell, SN: single-nuclei, WT: Whole Tissue, C: Cortex, G: Glomerulus, M: Medulla, IM: Inner Medulla, OM: Outer Medulla.

a Annotations were not available in the manuscript or its supplemental information, but a manual annotation based on reported markers was performed.

b Annotations were not available in the manuscript or its supplemental information, but annotations were retrieved from: https://github.com/hato-lab/kidney-endotoxin-sepsis-timeline-featureplot/blob/master/app.R.

Based on these evaluation results, we built an integration pipeline in which we first use a tuned (see STAR Methods) version of scVI to integrate all eight datasets. Second, we apply scANVI^24^^,^^25^ to the integrated latent space results from scVI together with cell type labels to refine the integration. We computed the same integration metrics as before for our tuned version of scVI-scANVI (Figure S1A). Notably, tuning scVI’s hyperparameters to maximize both batch separation and cell type similarity (see STAR Methods) improves the performance of scVI-scANVI considerably in both batch correction and biological conservation metrics.

After integration, the aligned compendium demonstrates that the different data sources have been properly aligned and no metadata is driving the differences observed in the UMAP space (Figures 1F and 1G).

### Integration highlights annotation inconsistencies across studies

**Figure 2:**
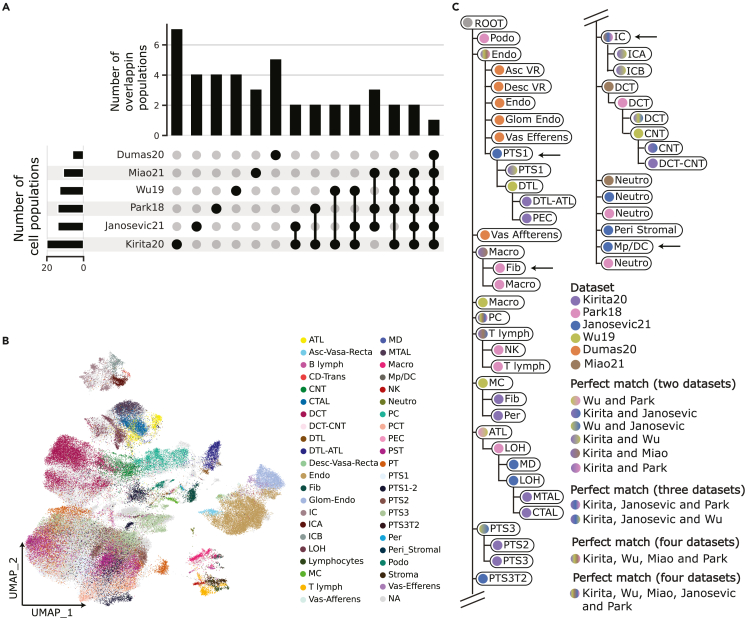
Learned classification tree from independently annotated datasets (A) UpSet plot visualizing the intersection and the number of common cell type annotations between the different datasets. Disconnected dots correspond to the number of unique cell types in each dataset, while connected dots represent the intersection between the datasets. Additionally, the number of cell types identified in each study is plotted alongside each dataset. (B) UMAP representation of the originally annotated cell types across all datasets. (C) Learned classification tree applying a k-Nearest Neighbor (kNN) classifier on the six annotated datasets. The color(s) of the tree nodes correspond to the supporting dataset(s) Missing populations: *PTS1*_*Janosevic21*_, *PTS2*_*Janosevic21*_. Arrows mark both inaccurate placements in the tree (*Fib*, *Stroma*) and cell types that can be further annotated to increase resolution *IC*: Intercalated Cell, *ICA*: Intercalated Cell Type A, *ICB*: Intercalated Cell Type B, *Endo*: Endothelial Cell, *Fib*: Fibroblast, *Macro*: Macrophage, *B lymph*: B lymphocyte, *Stroma*: Stroma cell, *NK*: Natural Killer, *T lymph*: T lymphocyte, *PT*: Proximal Tubule, *PTS1*: Proximal Tubule Segment 1, *PTS2*: Proximal Tubule Segment 2, *PTS3*: Proximal Tubule Segment 3, *PTS3T2*: Proximal Tubule Segment 3 Type 2, *PC*: Principal Cell, *PEC*: Parietal Epithelial Cell, *Per*: Pericyte, *DCT*: Distal Convoluted Tubule, *ATL*: Ascending Thin Limb of Henle, *MD*: Macula Densa, *LOH*: Loop of Henle, *CTAL*: Thick Ascending Limb of Henle in Cortex, *MTAL*: Thick Ascending Limb of Henle in Medulla, *CNT*: Connecting Tubule, *Podo*: Podocyte, *DTL*: Descending Thin Limb of Henle, *MC*: Mesangial Cell, *Neutro*: Neutrophil, *Asc-Vas-Recta*: Ascending Vasa Recta, *Desc-Vas-Recta*: Descending Vasa Recta, *Glom Endo*: Glomeruli Endothelial.

After integration, we investigated cell population annotations across the four datasets for which annotations were available or were manually annotated (Park18, Wu19, Kirita20, Miao21, Dumas20, and Janosevic21). The six datasets varied significantly in the resolution and the ontology used to annotate distinct cell populations. Only two cell populations were common between the five studies (Dumas20 only surveyed endothelial cells) based on the set of author’s annotated terms, with most of the annotations being dataset-specific (Figure 2A). For example, collecting duct intercalated cells (*IC*) can be further classified into type A (*ICA*) or type B (*ICB*), depending on the expression and localization of Slc4a1 in the membrane and the presence of a transport protein called pendrin, encoded by the *Slc26a4* gene. Whereas *ICA* cells lack pendrin and acidify the urine by excreting H^+^, *ICB* cells have pendrin, and secrete OH^−^ equivalents.^26^ Another example is proximal tubule cells (*PT*). While certain studies identify *PT* cells, some others further classify these cells in three different segments (*PTS1*, *PTS2,* or *PTS3*) depending on their location along the nephron (Figures 1A and 2B). These differences in ontology, together with the distinct annotation resolutions, highlight the subjective nature of unsupervised cell type annotation and the need for an integrated and comprehensive view of cell heterogeneity in the kidney.

To overcome these challenges, we used single-cell hierarchical progressive learning (scHPL),^20^ a method that automatically infers cell hierarchies from annotated datasets and builds a classification tree that can be used to classify unlabeled cells. We used scHPL to build a cell hierarchy and capture the relationships between kidney cell populations from the six annotated datasets. Perfect matches were found between cell populations across five (principal cells, *PC*), four (*Endothelial* and *Podocytes*), three (distal convoluted tubule, *DCT,* and *T lymphocytes*) and two datasets (*PTS1*, *PTS3*, ascending thick limb of Henle, *ATL*, *macrophages*, *ICA*, *ICB* and *CNT*). Not a single cell population matched across all six datasets (Figure 2C). On the other hand, some cell populations are misplaced in the tree. For example, the *fibroblasts* from Park18 (hereafter *cell type*_dataset_) are placed under *Macrophages*_Kirita20, Miao21_ another example are *PTS1*_Janosevic21_ cells which are placed under *Endothelial*_Kirita20, Wu19, Park18, Miao21_ cells. Other populations are lacking available resolution. For example, *IC*_Park18, Miao21_ cells, which appear as parent node of *ICA*_Kirita20, Wu19_ and *ICB*_Kirita20, Wu19_ cells. *Peri Stromal*_Janosevic21_ cells Moreover, some cell populations are missing in the final tree because they have been rejected (e.g., *PT*_Park18_ cells, *PCT*_Miao21_, *PST*_Miao21_, *PTS1*_*Janosevic21*_, and *PTS2*_*Janosevic21*_ cells could not be classified).

### Manual curation of annotations significantly improves hierarchy learning

To refine the cell tree constructed by scHPL and reduce the number of rejected populations, we performed a manual curation of the original cell population annotations (Figures S2–S4). The initial tree constructed by scHPL indicates that *Stroma*_Miao21_ cells have similar transcriptomic profiles to *T lymphocytes*_Park18_ (Figure 2C), which is supported by their overlap in the UMAP and the high similarity of their average expression profile (Figures S2A–S2C). This observation was supported by the expression of T lymphocyte markers^27^^,^^28^(*Cd4*, *Cd8a*, *Cd28*) in cells annotated as *Stroma* (Figure S2D). In addition, we compared the expression of *Cd4*, *Cd8a,* and *Cd28* in *Stroma*_Miao21_ cells, *T lymphocytes*_Park18_ and Kirita20 non-immune cell types (Figure S2E). As expected, *Stroma*_Miao21_ cells share the expression of these markers with *T lymphocytes*_Park18_ but not with non-immune populations. A similar scenario applies to *Fibroblasts*_Park18_, which are placed under the macrophages node (Figures 2C and S2E). We checked whether these cells might have been mislabeled by visualizing the expression of M1-M2 macrophage markers^27^^,^^29^(*Cd68*, *H2-Ab1,* and *Il4r*) (Figures S2F and S2G). We also plotted the expression of markers for all cell types present in the MKA in both *Stroma*_Miao21_ and *Fibroblasts*_Park18_ (Figure S2H). This confirmed that *Stroma*_Miao21_ mainly express T lymphocyte markers (*Cd247*, *Cd4* and *Cd8a*), whereas *Fibroblasts*_Park18_ express macrophage markers (*Cd68*, *H2-Ab1,* and *Cd74*). Based on these observations, we re-annotated *Stroma*_Miao21_ and *Fibroblasts*_Park18_ to *T lymphocytes* and *macrophages*, respectively.

We then evaluated the location of *PT* cells in the tree, which can be further classified in different segments (Segments 1, 2, 3, and 3 type 2; *PTS1*, *PTS2*, *PTS3*, *PTS3T2*). The proximal tubule is the first nephron segment after the glomerulus where numerous transporters regulate reabsorption and excretion.^5^ Janosevic21 specified the different *PT* cell types (i.e. *PTS1*, *PTS2*, *PTS3*, *PTS3T2*), while Park18 included the lower resolution term *PT* (Figure S3A) and Miao21 annotations included the terms proximal straight tubule (*PST*) and proximal convoluted tubule (*PCT*) (Figure S3B), Wu19 grouped *PTS1* and *PTS2* cells together (Figure S3C) and Kirita20 did not include *PTS3T2* (Figure S3D). To re-annotate these cells as *PTS1*, *PTS2, PTS3,* or *PTS3T2*, we used unsupervised clustering and visualized known markers to rename the resulting cell populations. The visualized markers were *Slc5a12*, *Cyb5a*, *Slc27a2,* and *Cyp7b1* for *PTS1*, *PTS2*, *PTS3,* and *PTS3T2*, respectively.^15^^,^^30^ In the case of *PTS1-2*_Wu19_, the population was matched to *PTS1*_Kirita20_ during training of scHPL. We re-annotated *PTS1-2*_Wu19_ as *PTS1*_Wu19_.

We refined the annotation of *IC*_Park18_, *IC*_Miao21_, and *Endothelial*_Park18_ cells following the same strategy as described above (Figsures S4A and S4B). *IC*_Park18_ and *IC*_Miao21_ cells were re-annotated as either *ICA* or *ICB* based on the expression of *Slc4a* (ICA marker) and *Insrr* (ICB marker) in the unsupervised clusters (Figure S4C). *Endothelial*_Park18_ cells were originally re-annotated as descending thin limb of Henle (*DTL*) by the original authors in the manuscript, but this correction was missing from the annotations provided with the dataset. Therefore, we similarly refined the annotation based on the expression of *Slc14a2* (DTL marker) and *Adgrl4* (Endothelial marker) (Figure S4D).

**Figure 3:**
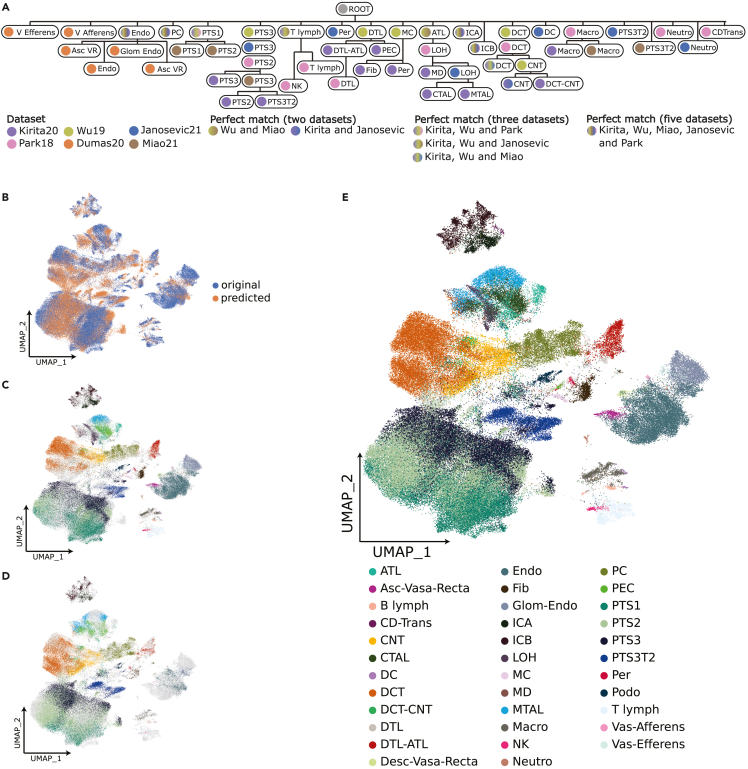
Hierarchically defined kidney mouse atlas (A) Learned classification tree on the four annotated datasets after manually harmonizing annotations. Tree nodes are colored by the supporting dataset or datasets in case of two or more cell populations matching. (B) UMAP plot visualizing cells used to train the classification tree (blue) and cells for which cell type was not known (orange). (C) UMAP representation of annotated cell typesPark18, Kirita21, Dumas20, Miao21, Janosevic21, and Wu19. (E) UMAP embedding of predicted cell types for Hinze20, Conway20 and cells labeled “unknown” or “missing.” e UMAP plot combining predicted and available annotations resulting in *the integrated mouse kidney atlas*. *IC*: Intercalated Cell, *ICA*: Intercalated Cell Type A, *ICB*: Intercalated Cell Type B, *Endo*: Endothelial Cell, *Fib*: Fibroblast, *Macro*: Macrophage, *B lymph*: B lymphocyte, *Stroma*: Stroma cell, *NK*: Natural Killer, *T lymph*: T lymphocyte, *PT*: Proximal Tubule, *PTS1*: Proximal Tubule Segment 1, *PTS2*: Proximal Tubule Segment 2, *PTS3*: Proximal Tubule Segment 3, *PTS3T2*: Proximal Tubule Segment 3 Type 2, *PC*: Principal Cell, *PEC*: Parietal Epithelial Cell, *Per*: Pericyte, *DCT*: Distal Convoluted Tubule, *ATL*: Ascending Thin Limb of Henle, *MD*: Macula Densa, *LOH*: Loop of Henle, *CTAL*: Thick Ascending Limb of Henle in Cortex, *MTAL*: Thick Ascending Limb of Henle in Medulla, *CNT*: Connecting Tubule, *Podo*: Podocyte, *DTL*: Descending Thin Limb of Henle, *MC*: Mesangial Cell, *Neutro*: Neutrophil, *Asc-Vas-Recta (Asc VR)*: Ascending Vasa Recta, *Desc-Vas-Recta (Desc VR)*: Descending Vasa Recta, *Glom Endo*: Glomeruli Endothelial, *V afferens*: Vas Afferens, *V Efferens*: Vas Efferens.

Following these annotation refinements, we applied scHPL to rebuild the cell tree and re-trained the classifier on the new tree. The new tree correctly captures the expected cell hierarchy, placing similar populations within the same node (Figure 3A). This is exemplified by the identification of the medullary and cortically thick ascending limb of Henle (*MTAL* and *CTAL*) as child nodes of loop of Henle (LOH) cells (Figure 1A). This shows the ability of the hierarchical model to group functionally and morphologically related cell types in the nephron. In this case, perfect matches between datasets were more easily identified, likely due to the lower number of rejected cells while training.

Next, we used the refined classification tree from scHPL (Figure 3A) to predict the cell type annotations for cells and nuclei from the remaining two unlabeled studies (Conway20 and Hinze20) (Figures 3B–3D). After merging the predicted and original labels, we obtained the final fully annotated adult healthy kidney atlas (MKA) (Figure 3E). The complete overview of the cell population shows that the integration process preserved the shared biological component between the different studies.

Due to the lack of labels for these two datasets, we could not perform a quantitative analysis of the obtained labels. To confirm our annotations, we visualized known markers for the major cell types in the nephron. Namely *PT*, *Podocytes*, *DTL*, *ATL*, *MTAL*, *CTAL*, *DCT*, *CNT*, *ICA*, *ICB,* and *Endothelial* cells (Figure S5A–S5D). Moreover, we compared the cellular composition of each dataset to that reported in the original studies. We found that the proportion of all predicted proximal tubule cells, i.e., *PTS1*, *PTS2*, *PTS3* and *PTS3T2*, matches the proportion described in the original publications, 77% in Conway20 and approximately 60% in Hinze20 (Figure S5E). The same applies to *MTAL*, *CTAL* and *DCT* in the MKA. These cell populations were described as *LOH/DCT* in Conway20 and *TAL* in Hinze20 with a proportion of approximately 7% and 10%, respectively (Figure S5E).

The MKA allowed us to annotate these datasets at a higher resolution than originally reported. For example, in Conway20 they annotated 15 cell types. We now identify 28 distinct populations, providing further resolution for annotations such as *LOH/DCT* (*MTAL*, *CTAL,* and *DCT* in the MKA) or *CD* (*CD-Trans*, *ICA*, *ICB,* and *CD* in the MKA). We also identify previously overlooked important cell populations, such as *PC* (Figure S5B). Another example is Hinze20, in which MKA identified 25 subpopulations among the original set of 10 cell types, including: *PTS1*, *PTS2*, *PTS3,* and *PTS3T2* instead of *PT*; *ICA,* and *ICB* instead of *CD-IC*; and *DTL* and *ATL* instead of *TL* (Thin Limbs) (Figure S5A). In summary, these annotations provide a comprehensive collection of cell types in the healthy kidney and are supported from at least one published dataset. Moving forward, we keep this resolution and cell type set. However, we could identify other cell types that were missing in the set of input annotations, such as vascular smooth muscle cells (Figure S6A). Unfortunately, we couldn’t find higher resolution populations for other cell types, such as macrophages. Markers for infiltrating monocytes did not show any expression pattern in the latent space that could indicate their presence, or any other subpopulations (Figure S6B).

Given the two different suspension types present in the MKA (i.e., single-cell and single-nuclei), we investigated whether there are sampling differences between them at the cell type level. Most cell types have an equal contribution of single-cell and single-nuclei datasets (Figure S7A). CNT, DCT-CNT, DTL-ATL, Fibroblasts, ICA, PC, Podocytes and PTS3T2 have a significantly higher contribution from single-nuclei datasets when compared to single-cell ones. We observed the biggest effect size in the PTS3T2 population. To understand if these differences are due to variability in sampling between single-cell and single-nuclei datasets, we compared the total number of detected PTS3T2 cells or nuclei in each of the datasets (Figure S7B). We observed that single-cell datasets had a very similar size in total to their single-nuclei counterparts, indicating that the lack of PTS3T2 cells in single-cell datasets is not due to overall under-sampling.

We sought to further explore the impact of suspension type in the cell types present in the MKA at the gene expression level. To this end, we correlated the batch-corrected expression for a given cell type between single-cells and single-nucleus (Figure S7C). All cell types had a significant correlation between cells and nucleus, with their correlation coefficient being higher than 0.65 in all of them.

### MKA accurately classifies unseen cells

**Figure 4:**
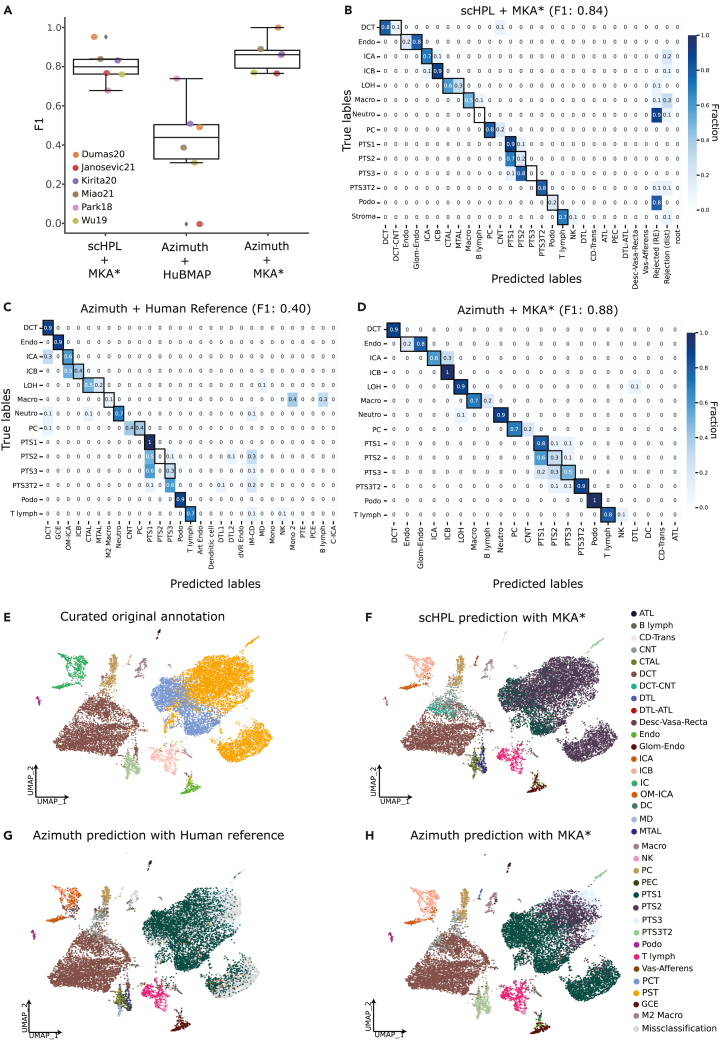
Evaluation of the scHPL classifier (A) Boxplot of median F1 scores (y axis) computed over 6-folds in three different scenarios (x axis). From left to right, scHPL trained with MKA∗, Azimuth’s label transfer using the HubMAP reference available^33^ and Azimuth’s label transfer using MKA∗ as reference. Each dot corresponds to the median F1 score computed across cell populations for a given training and validation set (i.e. MKA∗ and each of the annotated datasets in MKA respectively). (B–D) Confusion matrices normalized by class support size, computed using the predicted annotations by scHPL and our Atlas reference (B), the transferred labels from Azimuth’s human kidney reference.^32^ (C) or the transferred labels from Azimuth using our reference (D). Higher values indicate higher agreement between predicted and true cell labels. (E–H) UMAP plot of the Miao21 dataset colored by the original cell types (after manual re-annotation) (E), by the predicted cell types from the learned classification tree (F), by the transferred cell types from the Azimuth human reference (G) and by the transferred labels using Azimuth with our Atlas reference (H). *IC*: Intercalated Cell, *ICA*: Intercalated Cell Type A, *ICB*: Intercalated Cell Type B, *Endo*: Endothelial Cell, *Fib*: Fibroblast, *Macro*: Macrophage, *B lymph*: B lymphocyte, *Stroma*: Stroma cell, *NK*: Natural Killer, *T lymph*: T lymphocyte, *PT*: Proximal Tubule, *PTS1*: Proximal Tubule Segment 1, *PTS2*: Proximal Tubule Segment 2, *PTS3*: Proximal Tubule Segment 3, *PTS3T2*: Proximal Tubule Segment 3 Type 2, *PC*: Principal Cell, *PEC*: Parietal Epithelial Cell, *Per*: Pericyte, *DCT*: Distal Convoluted Tubule, *ATL*: Ascending Thin Limb of Henle, *MD*: Macula Densa, *LOH*: Loop of Henle, *CTAL*: Thick Ascending Limb of Henle in Cortex, *MTAL*: Thick Ascending Limb of Henle in Medulla, *CNT*: Connecting Tubule, *Podo*: Podocyte, *DTL*: Descending Thin Limb of Henle, *MC*: Mesangial Cell, *Neutro*: Neutrophil, *Asc-Vas-Recta (Asc VR)*: Ascending Vasa Recta, *Desc-Vas-Recta (Desc VR)*: Descending Vasa Recta, *Glom Endo*: Glomeruli Endothelial.

To evaluate the accuracy of cell type classification using MKA as a reference, we performed leave-one-dataset-out cross-validation experiments with two different classifiers. In the first experiment, we chose one of the annotated datasets as a test set and trained the scHPL classifier on the remaining datasets (hereafter MKA∗) at each iteration, with the same parameters as defined earlier in this manuscript. We then compared the performance of scHPL with Azimuth,^31^ a widely used pipeline to automatically annotate cells based on Seurat, in a second experiment. Following a similar approach as in the first experiment, we chose one of the datasets as a query dataset and set the rest of annotated datasets as our partial reference (MKA∗) and performed Azimuth’s workflow with default parameters. Finally, we evaluated the performance of Azimuth to predict mouse cell types using the available human ref.^32^ To do this, we performed a third experiment in a similar fashion to the previous two. At each iteration, we submitted each test set’s raw counts (namely Park18, Miao21, Kirita20, Wu18, Dumas20, and Janosevic21) as a query to the Azimuth web application using the human kidney reference. The median F1 score of all folds across the three experiments (Figure 4A), highlighted the importance of using the MKA when transferring labels to mouse datasets, regardless of the classifier. The human reference is also more likely to have outliers in terms of label transfer performance in a human-to-mouse scenario. For example, when using the human reference available, Park18 seems to be predicted at an accuracy closer to that obtained by using MKA (F1 score of 0.73). On the contrary, Janosevic21 is predicted with very poor accuracy (F1 score of 0.03).

To further highlight the value of the MKA, we tested the cell-type label transfer accuracy when using single-dataset references. Overall, the accuracy greatly depends on the query dataset for which we are trying to predict the labels (Figure S8), something that is mitigated by using the MKA as a reference (Figure 4A). Despite matching the predicted and original sets of labels to account for inconsistencies in annotation resolution (i.e., original labels included PTS1, PTS2, and PTS3 but the predicted labels from using a single-dataset reference include only PT), the accuracy of the predictions for a given query greatly depend on the reference used. An example of such case is Park18. Miao21 is the best reference in this case with other datasets quickly dropping in accuracy (F1 scores of 0.68, 0.46, and 0 for Wu19, Kirita21, Janosevic21, and Dumas20, respectively). Another important pitfall of using single-dataset references is exemplified by Kirita21. In this dataset, the authors define novel cell states that lay in-between known cell types (i.e., DCT-CNT or ATL-DTL) and annotate other cell types at a great resolution. These cell types are not captured by other datasets, which affects their performance as references when predicting labels from Kirita21.

In order to understand the contribution of different cell types to the overall F1 scores, we chose Miao21 as a test set and trained the scHPL classifier on the MKA∗. The resulting tree (Figure S9) was then used to predict the labels of cells from Miao21. Most of the original annotations from the dataset were accurately predicted by the scHPL classifier with median F1 score of 0.84 (Figures 4B and 4F). Moreover, scHPL further classified cells at a higher resolution than the low-resolution labels present in the original dataset (Figure 4E). For example, in the original study, Miao21 identified *LOH* cells, which scHPL can classify into *MTAL* and *CTAL*, the two major cell types present in the thick ascending limb of the loop of Henle. Notably, some cells and nuclei were assigned to the root node (i.e. unclassified). For example, *Neutrophils* were mostly rejected (Figure 4B). This is not surprising, since there were only 26 *Neutrophil* cells in the training data (i.e. MKA∗), which inevitably led to a poor performance in predicting *Neutrophils* in Miao21. On the other hand, one of the most abundant cell types in the training data (*DCT* with 4559 cells and nuclei) is correctly predicted 90% of the time (*DCT* and *DCT-CNT*). scHPL can reject cells due to the lack of cells from a specific population during training, e.g. neutrophils. But rejection can also mean that the query dataset includes novel cell populations not seen during training. In the latter case, rejected cells assigned can be further characterized and annotated to update the cellular knowledge stored in MKA.

As in our cross-validation experiments, we used Azimuth to predict the labels of our query dataset (Miao21) using both the human reference and our MKA∗. In the case of the human reference, despite having a wider array of cell populations (46 populations), Azimuth misclassified many cells with a median F1 score of 0.40 (Figures 4C and 4G). For instance, *PC* cells were classified as *CNT* or *DCT* 80% of the time. Previous studies have identified a transitional CNT-PC subpopulation of cells in healthy human kidney samples.^34^ This finding suggests that the mislabeled cells may not be a distinct cell type, but rather in a transitional stage, given their transcriptomic overlap. This is a high rate of misclassification considering that these are two very distinct cell types specialized in different functions in the nephron. These misclassifications can be due to the lack of a rejection option in Azimuth or differences in the cell type-specific transcriptomic profiles between human and mouse kidney, or a combination of both factors. When using our partial reference atlas (MKA∗) we were able to accurately classify cells in the query data with a median F1 score of 0.88 (Figures 4D and 4H). This result indicates that the low performance of Azimuth than scHPL is mainly due to the use of a human reference to classify mouse cells.

In order to understand how different populations contributed to the F1 score, we computed a median F1 score per cell type, model and for each fold in the cross-validation experiments (Figure S10). Two of the 14 populations included were accurately classified (F1 > 0.8) across the different validation experiments. In the case of the MKA∗+scHPL experiment, the number of accurately classified populations increases to seven out of 14. Despite proximal tubule cells (*PTS1*, *PTS2*, *PTS3,* and *PTS3T2*) being the most abundant cell type in the nephrons,^27^ we saw a lot of variation in the classification accuracy of these populations. In the training data (MKA∗), PT cells account for 45% of the total number of cells and nuclei. *PTS3T2* cells (3%) are accurately predicted when using MKA∗ and either scHPL or Azimuth. This can be explained by the lack of this population in the human kidney reference available. *PTS1* and *PTS2* cells (10% and 17%) display a high degree of F1 score variability across the different experiments (Figure S10). This is expected, as segments 1 and 2 can be identified morphologically but have almost identical functionality in the nephron.^35^ As a consequence, their transcriptomic profiles are highly overlapping, which has led to several authors considering them a single cell type.^11^^,^^14^ None of the reference and classifier combinations we tested accurately classifies both segments. *PTS3* is the least abundant cell type in the nephron and have the highest accuracy score when using the MKA∗ with Azimuth. Even in this case, 50% of the time *PTS3* cells are misclassified as either *PTS1* or *PTS2* (Figure 4D).

### Mouse kidney atlas facilitates the identification of robust cell population markers

Technological limitations in single-cell transcriptomics result in a high proportion of unmeasured genes leading to low replicability of cell type markers across different studies. We capitalized on the large collection of cells and nuclei from diverse samples in MKA to identify replicable cell population markers.

**Figure 5:**
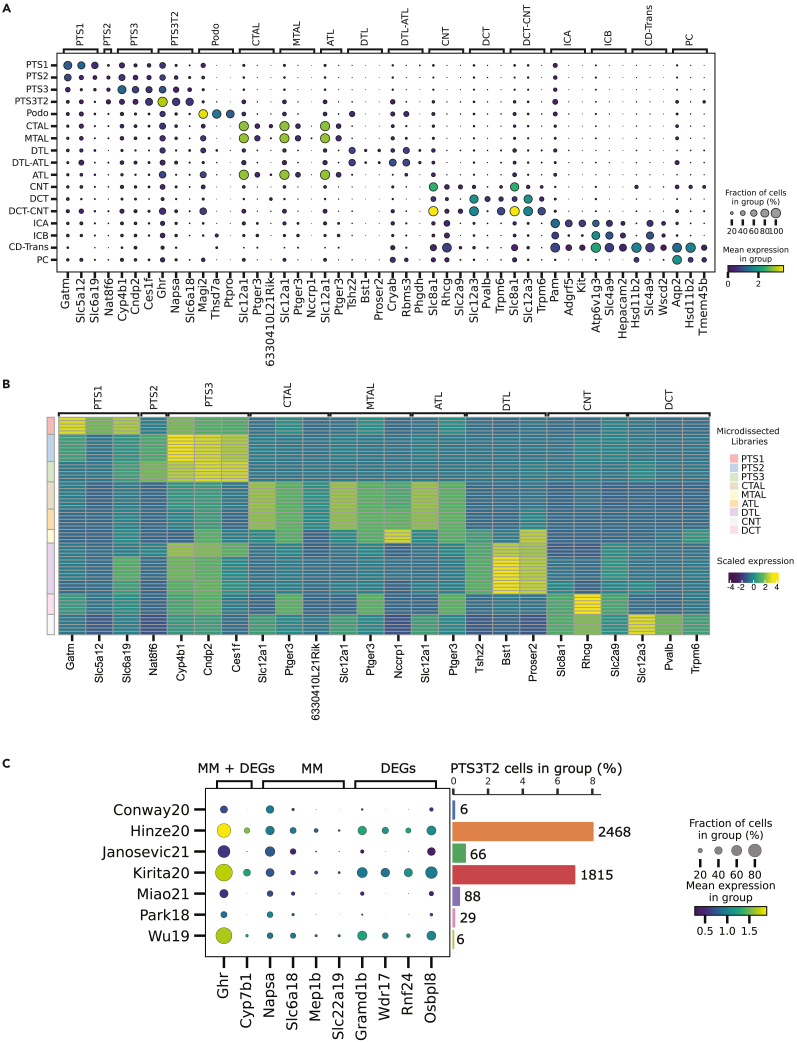
Joint downstream analyses highlight known cell type markers and help define meta-markers across studies (A) Dotplot of the top 3 meta-markers (when their recurrence is equal or greater than 2) per cell type across datasets. Values sorted by fold change and auroc. (B) Heatmap showing the scaled and normalized transcript per million (TPM) expressions of the top meta-markers in the micro-dissected kidney bulk-RNAseq libraries.^36^ Only matching cell types between the two experiments were kept. Columns represent the RNA-seq libraries, rows correspond to genes. Both rows and columns are annotated by cell type. In the case of rows, the annotation corresponds to the cell type from which these markers were identified in the atlas. For the columns, the annotations are the different regions from which the RNA-seq libraries are derived from. (C) Dotplot of the top 5 metamarkers (when their recurrence is equal or greater than 2; sorted by fold change of detection rate and auroc) and the top 5 DEGs for PTS3T2. Barplots show the number of PTS3T2 cells in each dataset both in relative (% of total cells in the dataset) and absolute terms (total number of cells on top of each bar). *IC*: Intercalated Cell, *ICA*: Intercalated Cell Type A, *ICB*: Intercalated Cell Type B, *Endo*: Endothelial Cell, *Fib*: Fibroblast, *Macro*: Macrophage, *B lymph*: B lymphocyte, *Stroma*: Stroma cell, *NK*: Natural Killer, *T lymph*: T lymphocyte, *PT*: Proximal Tubule, *PTS1*: Proximal Tubule Segment 1, *PTS2*: Proximal Tubule Segment 2, *PTS3*: Proximal Tubule Segment 3, *PTS3T2*: Proximal Tubule Segment 3 Type 2, *PC*: Principal Cell, *PEC*: Parietal Epithelial Cell, *Per*: Pericyte, *DCT*: Distal Convoluted Tubule, *ATL*: Ascending Thin Limb of Henle, *MD*: Macula Densa, *LOH*: Loop of Henle, *CTAL*: Thick Ascending Limb of Henle in Cortex, *MTAL*: Thick Ascending Limb of Henle in Medulla, *CNT*: Connecting Tubule, *Podo*: Podocyte, *DTL*: Descending Thin Limb of Henle, *MC*: Mesangial Cell, *Neutro*: Neutrophil, *Asc-Vas-Recta (Asc VR)*: Ascending Vasa Recta, *Desc-Vas-Recta (Desc VR)*: Descending Vasa Recta, *Glom Endo*: Glomeruli Endothelial.

Based on MKA, we identified *meta-markers*, which are genes that have a high detection rate and are reliable markers for a given cell population across different datasets (see STAR Methods for details). The resulting set of meta-markers per cell type included previously known markers (e.g. *Slc12a1* for both *MTAL* and *CTAL*, and *Slc12a3* for *DCT*), as well as novel candidates (e.g. *Bst1* for *DTL* or *Rhcg* for *CNT*) (Figure 5A and Table S2).

To verify our newly identified meta-markers, we verified their expression in the respective cell types using: (i) bulk gene expression data from micro-dissected samples and (ii) 10x Visium spatial transcriptomic data. First, we used the gene expression profiles of 64 bulk RNA-seq samples obtained from micro-dissected kidney segments generated by Chen et al.^30^ These segments (excluding *CD* and *Glomerulus*) are identified morphologically and ideally contain a single cell population each. We confirmed the expression of the top three meta-markers that appeared in at least two per cell population in the bulk RNA-seq samples. These correspond to *PTS1*, *PTS2*, *PTS3*, *CTAL*, *MTAL*, *ATL*, *CNT*, *DCT* and *DTL* (Figure 5B). Furthermore, we found a significant overlap between the MKA-based meta-markers and the microdissection-defined markers (Table S3). Second, we used 10x Visium spatial transcriptomic data of the healthy mouse kidney from GSE171406.^37^ We plotted the gene expression of two meta-markers for each of the following populations: *PTS3T2*, *DCT-CNT*, *DTL-ATL,* and *CD-Trans* (Figures S11A–S11D). *Napsa* and *Slc6a18* expression in the corticomedullary junction of the kidney co-localizes with what others have previously described as a *PTS3T2* cluster.^15^ *DCT-CNT (Slc12a3-Trpm6)* and *DTL-ATL (Cryab-Phgdh)* meta-markers follow a characteristic cortical and medullary expression pattern, respectively. This is expected given that the individual cell types are mainly localized in the cortex (*DCT* and *CNT*) or medulla (*DTL* and *ATL*). *CD-Trans* meta-markers (i.e. *Slc4a9* and *Wscd2*) display a heterogeneous pattern across the tissue slide. This suggests a location similar to *CD* and *IC* cells in the kidney. The low number of spots with detectable gene expression of both meta-markers is in agreement with the low fraction of cells labeled *CD-Trans* in MKA (less than 0.5%) and what others have reported.^8^

To highlight the value of MKA and the meta-markers we identified, we investigated rare, understudied cell populations. First, we characterized a recently described cell type, *PTS3T2* cells.^15^^,^^34^ Together with *PTS3*, *PTS3T2* cells are thought to play an important role in the kidney injury process.^33^ However, the few available marker genes for *PTS3T2* are based on unsupervised clustering of single-cell RNA-seq studies^15^ and are yet to be validated. Within our MKA-based meta-markers for *PTS3T2*, we identified previously known markers, such as *Slc22a13*, as well as novel markers: *Ghr* or *Mep1b* (Figure S10A). *Ghr* has been previously associated with chronic kidney disease,^38^ whereas *Mep1b* plays a role in acute kidney injury, with *Mep1b*^−/−^ mice showing improved renal function compared to WT mice.^39^ We compared the expression of the top five *PTS3T2* meta-markers with the top five *PTS3T2* differentially expressed genes in the MKA (Figure 5C and Table S4). Meta-markers, such as *Slc6a18* and *Napsa* displayed a robust expression pattern across the non-endothelial datasets (excluding Dumas20). However, DEGs, such as *Gramd1b*, *Wdr17*, *Rnf24,* and *Osbpl8* were expressed mostly at datasets with the highest number of *PTS3T2* cells, lacking replicability across studies. This was the case for the meta-markers *Mep1b* and *Slc22a19* too*. Ghr*, which encodes the growth hormone receptor was identified as both a meta-marker and a DEG with detectable expression in all datasets. However, *Ghr* is a significant DEG in 30 of the 36 cell populations included in the MKA (Table S2), indicating that *Ghr* expression is not specific. *Cyp7b1* is also identified as both a meta-marker and DEG but its expression pattern is biased toward Hinze20, Kirita20, and Wu19.

Although single-cell studies usually aim to describe discrete cell types, kidney’s nephrons are tubular structures formed by a continuum of epithelial cells. Due to this, cells with mixed transcriptomic profiles are likely to be sequenced.^9^ We set out to define meta-markers that are known for the cell types that are not only part of the mixed population, but also to identify novel markers of transitional cell types. In the case of *DCT-CNT* (Figure S11B), meta-markers included known markers for both *DCT*^30^ (*Slc12a3* and *Slc8a1*) and *CNT*^30^ (*Trpm6*) cells. Novel markers for this mixed population included *Acss3* and *Ltc4s*.

*Cryab*, a known marker for *ATL* and *LOH* cells, is identified as a meta-marker for *ATL-DTL* cells (Figure S11C). Others not previously known meta-markers include *Rbms3*, *Phgdh,* and *Slc4a11*. Some of these genes have already been implicated in the kidney biology. For instance, *Phgdh* has been identified as a treatment target in kidney cell carcinoma in patients resistant to HIF2α antagonists.^40^ *Slc4a11* is known to be expressed in *DTL* cells, although expression has been described only in the medullary part of the kidney.^41^

Next, we investigated the novel collecting duct transitional cell population (*CD-Trans*), which was described by Park and colleagues^8^ and by Chen et al.^36^ who labeled these as “hybrid cells”. *CD-Trans* cells have been described as an intermediate state between *PC* and *IC* cells, expressing markers for both cell types.^8^^,^^36^ While *ICA* and *ICB* cells play a role in the regulation of acid-base homeostasis,^35^ *PC*s main function is salt and water transport. In the latter case, sodium (epithelial sodium channel, *Scnn1a/b*) and water (Aquaporin 2, *Aqp2*) channels control the levels of Na^+^ and K^+^ in plasma, blood pressure, and extracellular fluid osmolality. Further understanding of *CD-Trans* cells has been hampered by their low abundance in the kidney, often being masked by other cell types, such as proximal tubule cells. In MKA, *CD-Trans* cells were identified in four datasets (Park18, Miao21, Janosevic21, and Conway20) after annotation of the full atlas with 60 cells in total. Our meta-marker list for *CD-Trans* cells includes *Hsd11b2*, *Slc4a9*, *Wscd2,* and *B3gnt7* which were found to be highly accurate and able to confidently classify cells as *CD-Trans* (Figure S11D). Kidney-specific *Hsd11b2*^−/−^ mice show systemic salt-dependent hypertension.^42^ Moreover, *CD-Trans* cells in the MKA express both *Aqp2* and *Slc4a9* (meta-marker for *ICB*), further confirming the transitional state between *PC* and *IC* of these cells.

## Discussion

The maturity of single-cell and single-nuclei transcriptomics becomes apparent by the ever-increasing number of publications applying these technologies.^43^^,^^44^ Although this has given rise to a vast collection of publicly available cellular transcriptomes, researchers continue to analyze their work in an isolated environment, often without considering the data from other reports. As it has been recently noted in the literature,^34^ the relationships between the populations defined in kidney single-cell studies are not clear and integrative studies are needed. Here, we integrate cells and nuclei from eight independent studies (Table 1) to create the first mouse kidney atlas. We demonstrate that, despite between-sample biological and technical differences, our atlas establishes a robust and comprehensive view of the cell heterogeneity present in the mouse kidney.

A major challenge in single-cell analyses is cell type annotation. Usually, cell types are annotated based on the expression of marker genes in unsupervised clusters. Clustering algorithms require the tuning of hyperparameters, leading to a subjective choice on the number of clusters. This is aggravated by the possible presence of new (sub) cell types in the dataset, which usually causes over-clustering.^45^ This introduces subjectivity to the analysis, ultimately leading to incomplete and ambiguous annotations between studies. We highlight these inconsistencies in the case of the mouse kidney using scHPL, a supervised hierarchical machine learning model. By refinement of these annotations and further cell type learning, we improve the atlas reference transcriptome, accurately capturing consensus cell identities across studies. An important feature of such a model is its ability to capture the different resolutions at which cell types have been annotated. For example, some studies limit their labeling to *LOH* cells while others further classify these cells as *MTAL* or *CTAL*.^9^^,^^14^ In our work we convey a hierarchically defined atlas, further characterizing the variety of cell types present in the healthy mice kidney (Figure 3E). In consequence, we identify 35 distinct cell types, including both high- and low-resolution annotations. We have shown that most of these cell types are equally detected in both single-cell and single-nuclei studies (Figures S7A and S7B). Despite single-cell studies having a similar number of cells, PTS3T2 cells are detected in higher proportions in single-nuclei studies. We hypothesize that PTS3T2 cells are harder to detect in single-cell studies, possibly due to differences in their survival in cell and nuclei isolation protocols. Differences in cell type composition between single-cell and single-nuclei studies have been reported before.^46^ As noted by Wu et al., single-nuclei RNA-seq was able to detect 20-fold more Podocytes than the proportions reported by single-cell studies. In addition, mesangial cells were completely missing from their single-cell dataset, further elucidating the differences in detection between dissociation protocols.

Unfortunately, our atlas cannot predict, with full accuracy, all cell types in the kidney. This limitation is not exclusive to this organ, as supervised cell classification remains a challenge for all tissues. It is often due to the lack of a precise definition of cell types, lack of robust markers, technical limitations, and sampling variability.^46^ In addition, renal plasticity and the ability of renal cells to switch cell type might generate some less defined cells.^47^^,^^48^ In our work, we highlight the common misclassification of *PTS1*, *PTS2,* and *PTS3* cells by different methods (Figures 4B–4D). Although functional differences between the segments are known, the different segments have traditionally been identified based on cell ultrastructure.^49^ This results in their transcriptomes being too similar, rendering these cells hard to classify computationally. As indicated by Shanley and colleagues,^50^ the third segment of the proximal tubule is particularly vulnerable to ischemic damage. It is not yet clear whether what we and others^15^ have identified as *PTS3T2* constitutes a genuine cell type or rather a damaged state of *PTS3* cells. We would like to note; however, that by extensive integration of datasets we can largely overcome these shortcomings, as we have demonstrated in the present work. As the field develops, and clearer definitions are proposed, the inclusion of more datasets into our atlas will further enhance cell type identities and classification. For example, if including larger unannotated healthy samples in the MKA results in more cells being classified as *PTS3T2*, there would be *in silico* evidence that this cell type is indeed present in healthy proximal tubules. However, to confirm its identity as a cell type, a complete understanding of its origin from a developmental perspective is probably needed.

The importance of our work is further highlighted by the pressing need to develop novel therapies for kidney failure. Kidneys are the most frequently transplanted organ. Due to the increasing prevalence of chronic kidney diseases in the population, demand exceeds the number of available donors. And strategies based on renal (stem) cells are being investigated. On grounds of these and other shortcomings, as noted in the literature,^51^ an understanding of the cell heterogeneity present in the kidney is needed in order to develop much-needed therapies. The efficacy of these will depend on the cell type-specific expression and activity of pathways.^52^ Despite this, the knowledge of cell types, its markers and the molecular mechanisms and pathology underlying these diseases at the single-cell level is still incomplete. For example, a recent study shows that *CNT* cells can display a partial *DCT* phenotype.^53^ However, this transitional cell type (*DCT-CNT*) is usually not identified or masked by more abundant cell types in single-cell studies. Consequently, most reports identify individual *CNT* and *DCT* clusters.^54^ To this end, the kidney atlas can aid the discovery of robust novel markers for *DCT*-*CNT* cells. These markers are detected across the different datasets and can accurately classify *DCT*-*CNT* cells. As demonstrated by the above example, we identify meta-markers for the cell types present in our atlas, including previously known and novel genetic markers. When compared to markers obtained without accounting each individual dataset in an integrated space, meta-markers with a high detection rate can provide replicability that generalizes the cell type identities defined in our atlas. A clear example of such a scenario is *DTL-ATL* cells. As has been described previously, one of the meta-markers identified for this population is *Slc4a11* which expresses a membrane transporter involved in water, ammonia, and H^+^ transport. Its expression has been located in DTL cells within the outer medulla and the outer stripes of the inner medulla in mice.^41^

These findings will benefit the broader kidney research community, for example, by aiding the robust *in vivo* identification of cell types. Since human and mouse kidneys show important physiological differences at the cellular level,^55^ we believe our work is especially relevant in mouse models. The discovery potential of our atlas; however, is much broader and largely not explored. We acknowledge that, although statistically robust and *in silico* verified with micro-dissected nephron segments^30^ and spatial transcriptomics tissue slides,^37^ these compendium-wide markers need further *in vivo* validation.

Cellular knowledge of the kidney is likely to change in the coming years. As technologies improve and innovative studies are published, novel cell types will be described. Likewise, cell identities will be re-defined in newer contexts. We aim to incorporate these changes within the atlas in a continuous fashion. We provide a learnt transcriptome-based cell hierarchy that can be easily updated and improved with newer studies, updating the cellular knowledge captured in the compendium. In addition, our atlas is missing cell types that were not present in the original set of annotations provided by the authors. We’ve shown that the MKA can potentially detect previously unannotated cell types, such as vascular smooth muscle cells. Because we used scVI and scANVI as our integration model, we can leverage scAarches^56^ to update the latent space of our atlas without retraining. For example, the recently published dataset by Song et al.,^57^ could be used to enhance the MKA with rich immune annotations. In other instances, updating the latent space and scHPL’s classification tree will allow us to annotate matching cell types and identify potential novel populations that arise from treatment or disease state. To account for the technical variation of new datasets in the context of the MKA, one can make use of the pre-trained and optimized model, we present to obtain an updated latent space that we then use to update the classifier. To make this easily accessible to the community, we share our atlas via a user-friendly web interface, hosted at cellxgene (https://cellxgene.cziscience.com/e/42bb7f78-cef8-4b0d-9bba-50037d64d8c1.cxg/).

In summary, we leverage the large collection of publicly available single-cell and single-nuclei studies and establish a dynamic atlas of the mouse kidney. We demonstrate the extraordinary power of such approach by providing robust markers for elusive cell types. However, the full potential of the created compendium is yet to be explored.

### Limitations of the study

This is the first complete single-cell and single-nuclei atlas of the mouse healthy kidney that harmonizes annotations across several publicly available datasets. However, there are several limitations in our study. Firstly, the lack of *in vitro* or *in vivo* validation of the computed metamarkers. Secondly, lack of more recent studies and different single-cell technologies. All the cells and nuclei in our atlas come from droplet-based libraries and short read sequencing. Adding plate-based technologies in the atlas might prove beneficial in the future, as more low-abundant transcripts are detected, a more accurate cell type classification will be achieved.

## STAR★Methods

### Key resources table

**Table:** 

REAGENT or RESOURCE	SOURCE	IDENTIFIER
**Software and algorithms**		

Python 3.7.12	Python	python.org
R 4.0.5	R	r-project.org
scvi-tools 0.19.0	GitHub	github.com/scverse/scvi-tools (0.19.0)
scHPL 1.0.0	GitHub	github.com/lcmmichielsen/scHPL (1.0.0)
Custom code	This paper	github.com/nrclaudio/MKA

**Datasets**		

Wu19	https://doi.org/10.1681/ASN.2018090912	GSE119531
Miao21	https://doi.org/10.1038/s41467-021-22266-1	GSE157079
Park18	https://doi.org/10.1126/science.aar2131	GSE107585
Kirita20	https://doi.org/10.1073/pnas.2005477117	GSE139107
Dumas20	https://doi.org/10.1681/ASN.2019080832	E-MTAB-8145
Conway20	https://doi.org/10.1681/ASN.2020060806	GSE140023
Hinze21	https://doi.org/10.1681/ASN.2020070930	GSE145690
Janosevic21	https://doi.org/10.7554/eLife.62270	GSE151658

### Resource availability

#### Lead contact

Further information and requests for analyses or method details will be fulfilled by the lead contact, Ahmed Mahfouz (a.mahfouz@lumc.nl).

#### Materials availability

This study did not generate new unique reagents.

### Method details

#### Collecting raw data and quantification of reads

All raw fastq files were downloaded from the Sequence Read Archive (SRA) using *parallel-fastq-dump* (v0.6.7; https://github.com/rvalieris/parallel-fastq-dump). Accession numbers and other relevant metadata are provided in Table 1. Single-cell and single-nuclei droplet-based sequencing data were aligned and quantified using kallisto/bustools^58^^,^^59^ (kb-python v0.26.4) *ref* and *count* wrappers, specifying --workflow nucleus in the case of single-nuclei sequencing experiments. Reads were pseudo aligned to the mouse reference genome GRCm38 downloaded from Ensembl.^60^

#### Pre-processing of sequencing data and normalization

Filtered count matrices from Kallisto/bustools were used when the cell count was within a 10k margin from the matrices deposited by the authors. Otherwise, the unfiltered count matrices were loaded, and barcodes were matched between the author’s and the unfiltered set of cells. Count matrices were pre-processed using Scanpy^61^ (v1.8.1). We applied quality filters to all samples, specifically, we filtered out cells with more than 50% of counts derived from mitochondrial genes. Furthermore, we applied dataset-specific quality filters based on the number of detected genes. These filters are available in Table S5. Samples were merged and normalized for plotting purposes with Scanpy’s normalize_total.

#### Integration benchmark

We compared several integration methods for our use case, including scVI, scANVI,^24^^,^^25^ Harmony, Scanorama,^21^^,^^23^ and Seurat’s.^22^ Based on the evaluation results (see below in the results section), we chose to use a hybrid approach in which we start with fully unsupervised integration using scVI followed by a refinement step using scANVI (Figure S12, steps 1–2). scANVI uses cell type labels to inform the manifold-learning process such that cells with the same label are explained by similar low-dimensional features. This improves the representation learnt by scVI by incorporating biological information (such as cell types) in the model. This workflow (i.e. improving the latent representation of scVI with cell type labels using scANVI) is denoted as scVI-scANVI from now on. As different hyperparameter combinations and model configurations can affect the performance of deep learning models, we used Ray tune^62^ to optimize scVI’s model. Raw counts and batch information were used to test 1000 different hyperparameter combinations. Our search space consisted of model configurations such as continuous and categorical covariates; model hyperparameters such as dropout rate, number of layers and number of latent dimensions; learning hyperparameters such as learning rate and pre-processing steps such as highly variable genes (HVG) filtering and number of HVGs. The objective function to optimize was the silhouette score of both batch and cell type information as implemented in scib.^63^ Detailed information and the scripts used to perform these analyses are available at https://github.com/nrclaudio/MKA.

#### Integration metrics

Batch and biological conservation metrics were computed using scib (v1.0; https://github.com/theislab/scib). Note that some of the metrics are scaled to range from 0 to 1, for details refer to the original publication.^63^ Batch conservation metrics include graph Local Inverse Simpson’s Index^21^ (LISI), kBET, Average Silhouette Width (ASW) and Principal Component Regression (PCR).^64^ In short, these metrics quantify the alignment between the different batch labels in the data. Specifically, kBET examines to what extent the different batches are mixed when neighborhoods are randomly sampled, LISI captures the diversity of batches within a local neighborhood of cells and PCR explains the total variance attributed to the batch variable when regressed on the Principal Components of the data. Biological conservation metrics include some of the previous metrics applied to cell type labels (cell-type ASW and LISI), Adjusted Rand-Index (ARI),^65^ Normalized Mutual Information (NMI),^66^ trajectory, cell cycle and variable gene conservation. These metrics quantitatively assess how much of the original biological variation is kept in the integrated space. The final score for each evaluation was computed as a weighted average of biological conservation and batch removal scores, with weights 0.6 and 0.4 respectively.

#### Dataset integration

We used the method with the highest overall score in the benchmark to integrate the different studies (i.e. our tuned version of scVI-scANVI). The hyperparameter configuration with the highest silhouette score obtained in our tuning experiment (see Integration benchmark) was then used to train scVI. In our case, we reduced the feature space of our atlas to the top 3000 HVGs. Variable genes were obtained using Scanpy’s highly_variable_genes with the flavor set to seurat_v3 and the batch_key set to the different datasets of origin. We included the percentage of mitochondrial reads as a continuous covariate in the model and the source of the material (cells or nuclei) as a categorical covariate. The model was initialized with 2 hidden layers, 26 latent dimensions, a dropout rate of 0.096 and the gene likelihood set to a Negative Binomial distribution. The model was then trained for a total of 111 epochs with a learning rate of 0.0013. The obtained model was then used as input for scANVI in order to further improve the latent space representation. We included available cell type annotations and set the unlabeled_category to the set of cells with missing annotations. The scANVI model was trained to a maximum of 20 epochs and with 100 cell subsamples per label class per training epoch.

#### Dimensionality reduction

After integration and batch -correction, 26 latent dimensions were obtained from the model. These were used as input for the Nearest Neighbor graph calculation using Scanpy’s neighbors function. We further reduced the dimensionality to visualize the data in a 2D UMAP using 26 latent dimensions.

#### Similarity metrics

To assess cell population similarity across studies, pairwise similarity measures were computed using sklearn^66^ (v0.23.2) pairwise_distances with the correlation metric. The similarity between two cell populations is reported as 1 – correlation distance between their average normalized transcriptomic profile.

Correlations between single-cell and single-nuclei profiles were computed using scipy’s pearson r (scipy.stats.pearsonr). The input vectors per cell type and suspension type type were obtained using scANVI’s get_normalized_expression with the transform_batch option set to the list of datasets in the atlas. The counts were then scaled by a factor of 1000.

#### Cell type learning and classification

All 26 latent dimensions from the annotated datasets (Park18, Wu19, Miao21, Kirita20, Dumas20 and Janosevic21) along with their original (Figure S12 step 3) or curated (Figure S12 step 4) cell type labels were used as input for single-cell Hierarchical Progressive Learning^20^ (scHPL, v1.0.0). For both the original and the curated labels, the classification tree was learnt using a kNN and default values. To classify the cells that were missing annotations, the learnt tree and the latent dimensions from Hinze20 and Conway20 were used as input for scHPL’s predict_labels function.

#### Evaluation of the classifier

We used leave-one-dataset-out cross-validation experiments to evaluate the classifiers performances. At each iteration we select one of the six datasets as a test set and treat the rest of the dataset as our training set.

To evaluate the performance of scHPL, the classification tree was learnt as described in the previous section. At each iteration, the test set labels were predicted and compared to the original curated labels. To measure the accuracy of the prediction, the F1 score (harmonic mean of the precision and recall) was computed, for every cell population, using scikit-learn v1.0.1 f1_score function with the average set to micro. The overall F1 score per dataset were computed as the median of F1 scores across cell populations.

To compare the performance of scHPL trained in our reference with other methods and references, we submitted each test set’s raw count data as a query to Azimuth with the Human Kidney Reference atlas.^31^^,^^32^ We kept the quality control filters we applied in our own pre-processing. The *l2.annotation* labels were transferred to the query using Azimuth. We also tested the performance of Azimuth’s workflow (In Seurat^22^: Seurat::SCTransform, Seurat::FindTransferAnchors and Seurat::MapQuery) to transfer the labels from our reference to the test query dataset. As described previously, the annotations predicted by Azimuth with the Human Kidney Reference and our own reference were compared to the original curated labels of the query. The accuracy of this prediction was computed as an overall F1 score.

For each evaluation experiment (i.e. scHPL trained with our reference, Azimuth trained with our reference and Azimuth trained with the available Human reference) the median F1 score across all folds was computed.

In the case of Miao21, for each pair of predicted and original labels, confusion matrices were computed using scHPL’s confusion_matrix function. The row vectors of these matrices were normalized to sum up to 1.

To compare the label-transfer accuracy of using single-dataset references against using our atlas as a reference, we performed evaluation experiments on each of the studies in our atlas that have available annotations (i.e. Miao21, Park18, Kirita21, Janosevic21, Wu19 and Dumas20). For each dataset, we predicted the original labels using Azimuth’s label transfer workflow treating each of the remaining studies as a reference. For example, in the case of Miao21, we predicted its labels from five different references, corresponding to each of the remaining datasets (i.e. Park18, Kirita21, Janosevic21, Wu19 and Dumas20). We then computed a median F1 score across references for a given query.

#### Differential gene expression analyses, meta-marker discovery and verification

Cell type markers were computed using Scanpy’s rank_gene_groups function with the Wilcoxon rank-sum test. Meta-markers were computed using the MetaMarkers R package^67^ (v0.0.1; https://github.com/gillislab/metamarkers). Raw counts were converted to CPM values (as in original work). Markers were computed for each dataset with compute_markers to then obtain meta-markers using make_meta_markers. These two functions are using a Mann-Whitney test per dataset and an aggregation based on meta-analysis of the obtained p-values, respectively. Pareto boundary markers (i.e. markers with high precision and detection rate) were visualized using plot_pareto_markers.

#### In silico verification of meta-marker expression in kidney tissue

To verify that the expression of the computed meta-markers agrees with the spatial location of their cell types, we plotted their log-normalized gene expression values in a healthy mouse kidney spatial transcriptomics tissue slide^37^ from Gene Expression Omnibus (GEO, GSE171406). Spots with less than 2000 unique genes expressed or higher than 50% of mitochondrial reads were removed.

#### Comparison with microdissected kidney bulk RNA-seq

TPM values were downloaded from GSE150338. From a total of 96 cell type bulk RNA-seq libraries, we kept 64 corresponding to the matching cell types in our atlas. TPM values were normalized as log2(TPM + 1). We then visualized the normalized expression of the previously computed cell type markers in the bulk RNA-seq context using pheatmap (https://github.com/raivokolde/pheatmap).

To test the significance of the overlap between the lists of differentially expressed genes (i.e. cell type markers defined by either our kidney atlas or the microdissection study) we used scipy’s v1.5.4 Fisher exact test (fisher_exact) in every cell type present in both the atlas and the microdissection study. We used the list of genes present in the atlas as background in the test. In both cases we considered as significant those genes with an adjusted (using Benjamini-Hochber’s FDR correction) p-value < 0.01. 99% Confidence intervals were computed for the odds ratios obtained in the test. This test evaluates whether a list of significant markers is independent of the list of markers that it is being compared to.

## Data Availability

• All single-cell and/or single-nuclei RNA-seq datasets used in this study are publicly available. Their accession numbers are listed in the key resources table. • Jupyter notebooks and scripts used in the analyses as well as supplemental data are available on Github (https://github.com/nrclaudio/MKA). Interactive visualization and downloading of the kidney mouse atlas are available at cellxgene (https://cellxgene.cziscience.com/e/42bb7f78-cef8-4b0d-9bba-50037d64d8c1.cxg/). • Any additional information required to reanalyze the data reported in this paper is available from the lead contact upon request.

## Supplemental information

Document S1. Figures.S1–S12

Table S1. Integration metrics, related to Figure S1

Table S2. Differential gene expression results for all cell types in the MKA, related to Figure 7

Table S3. Test statistics for the Fisher exact test between the markers defined in the MKA and micro-dissected nephron segments, related to Figure 5

Table S4. List of identified meta-markers per cell type together with AUROC and fold change of detection rate obtained from the MKA, related to Figure 7

Table S5. Quality filters per dataset, related to STAR Methods
