# Supplementary material for: A comprehensive mouse kidney atlas enables rare cell population characterization and robust marker discovery
Source: iScience. 2023 May 18;26(6):106877. doi: 10.1016/j.isci.2023.106877 (PMC10238935; doi:10.1016/j.isci.2023.106877)
Supplement: Document S1. Figures.S1–S12 [file mmc1.pdf]

iScience, Volume *26*

Supplemental information

A comprehensive mouse kidney atlas enables rare cell population characterization and robust marker discovery

Claudio Novella-Rausell, Magda Grudniewska, Dorien J.M. Peters, and Ahmed Mahfouz

Supplementary Figure 1

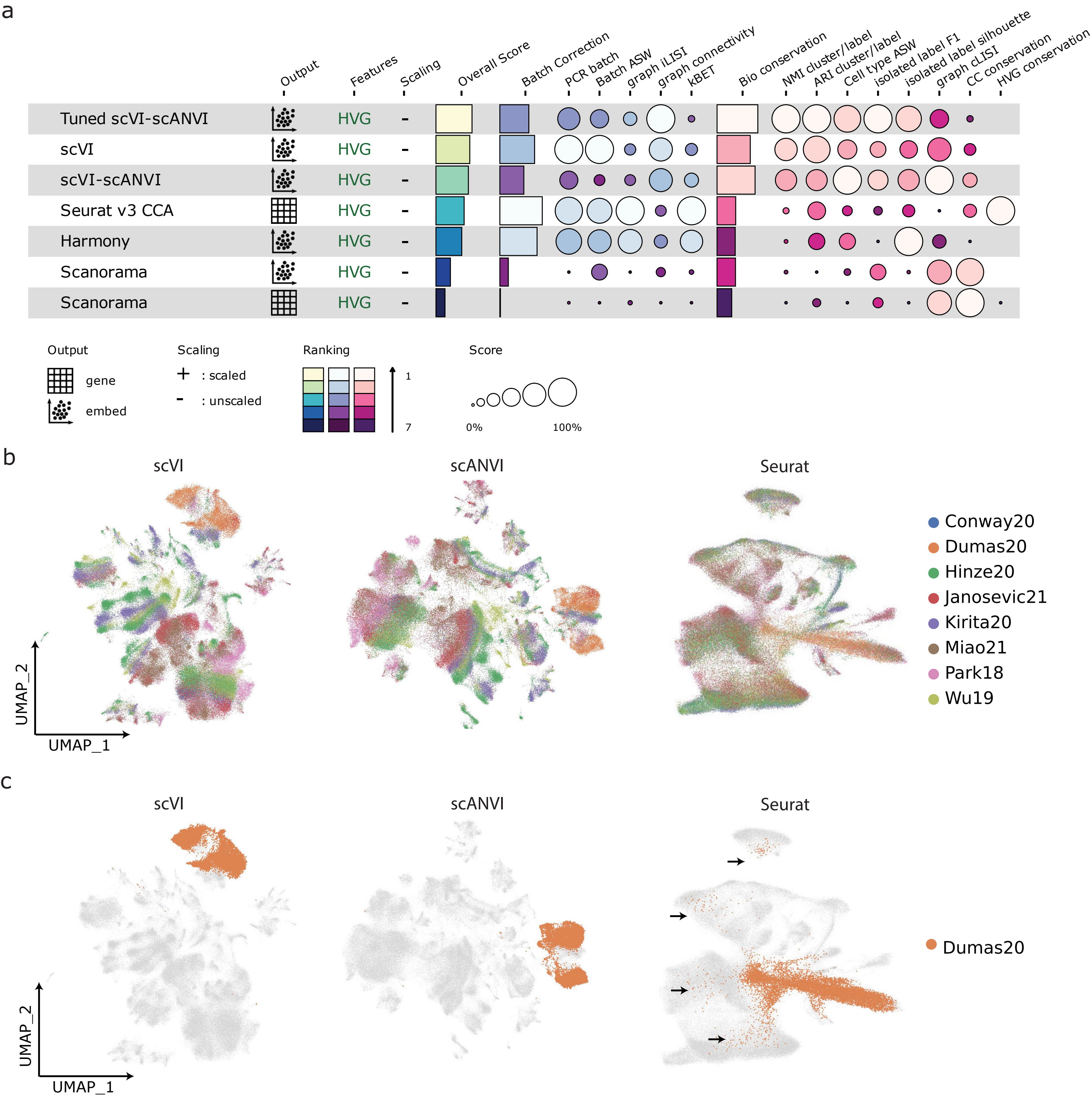

Figure S1. Comparison of integration methods. (a) Integration benchmark summary plot generated using default parameters for all methods compared. In all cases, 3000 highly variable genes were selected before integration. Methods are ranked based on overall score, computed with a weight of 0.6 and 0.4 for biological conservation and batch correction scores respectively (See Methods for details). **(b)** UMAP plots generated using batch-corrected latent features (scVI and scANVI) or batch-corrected expression matrices (Seurat). Coloured by dataset of origin. **(c)** UMAP plots of the different integration methods highlighting in orange cells from the Dumas20[13] dataset, containing exclusively endothelial cells. Arrows roughly indicate cells whose signal is overcorrected for batch differences, diluting the biological signal coming from the Dumas20 dataset.

Supplementary Figure 2

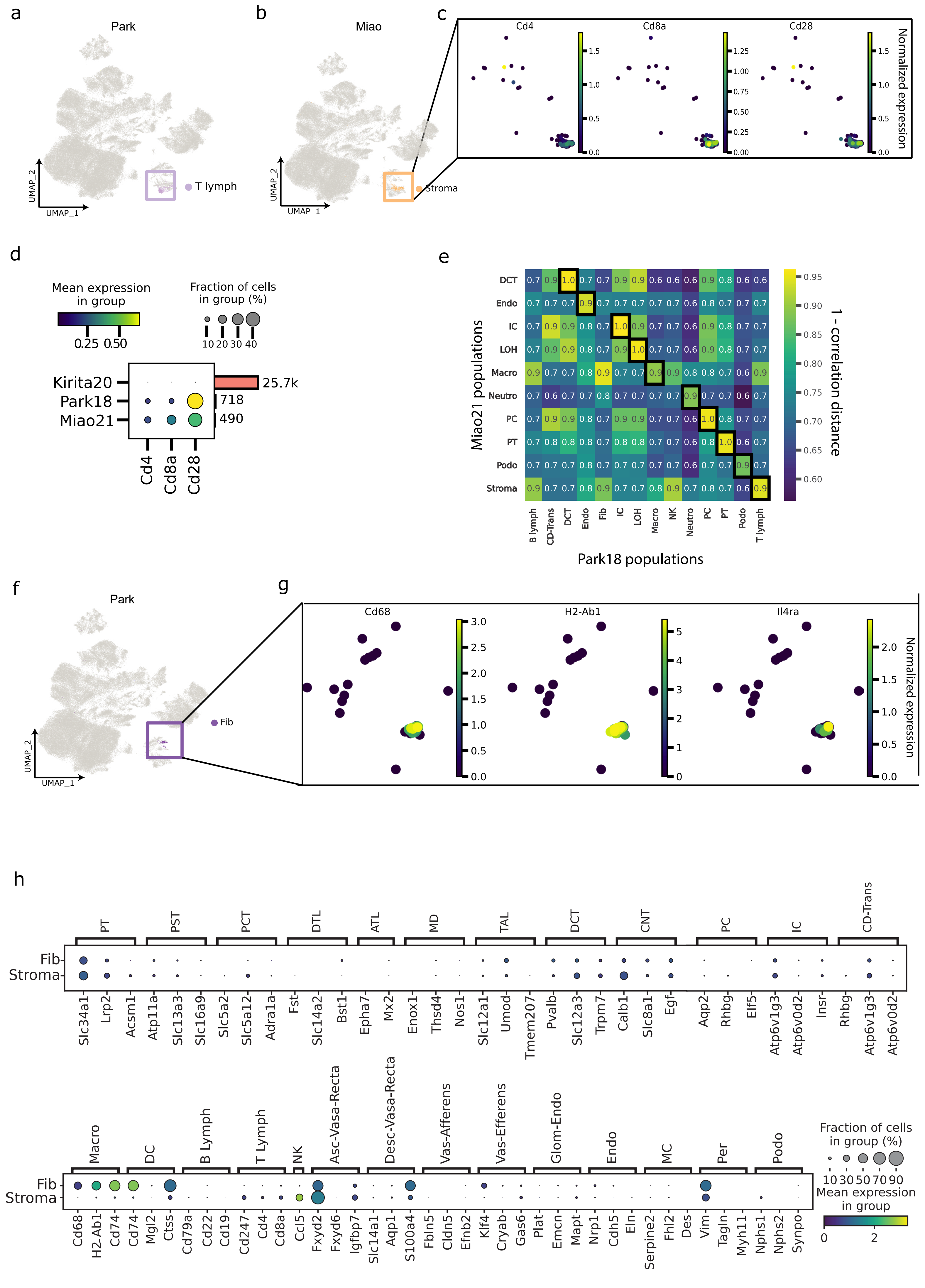

Figure S2. Manual annotation of dissenting cell populations based on scHPL. (a-b) UMAP plots, coloured by both *T lymphocytes*$_{\text{Park18}}$ (a) and *Stroma*$_{\text{Miao21}}$ cells (b). **(c)** UMAP plots of *Stroma*$_{\text{Miao21}}$ cells coloured by scaled *T lymphocyte* marker expression (*Cd4*, *Cd8a*, *Cd28*). **(d)** Dot plot showing the frequency and average level of expression in selected *T lymphocyte* markers (*Cd4*, *Cd8a*, *Cd28*) in *Stroma*$_{\text{Miao21}}$ cells, *T lymphocytes*$_{\text{Park18}}$ as positive control, and Kirita20 non-immune cells as a negative control. The bar plot (right) shows the total cell/nuclei count in each row. **(e)** Heatmap of the similarity (expressed as 1 – correlation distance) of cell populations between Miao21 and Park18 **(f)** UMAP plot, coloured by *Fibroblasts*$_{\text{Park18}}$. **(g)** UMAP plots of *Fibroblast* cells coloured by scaled *Macrophage* marker expression (*Cd68*, *H2-Ab1*, *Il4ra*). (h) Dotplot of canonical markers[44] per cell type in both *T lymphocytes*$_{\text{Park18}}$ and *Stroma*$_{\text{Miao21}}$ cells.

Supplementary Figure 3

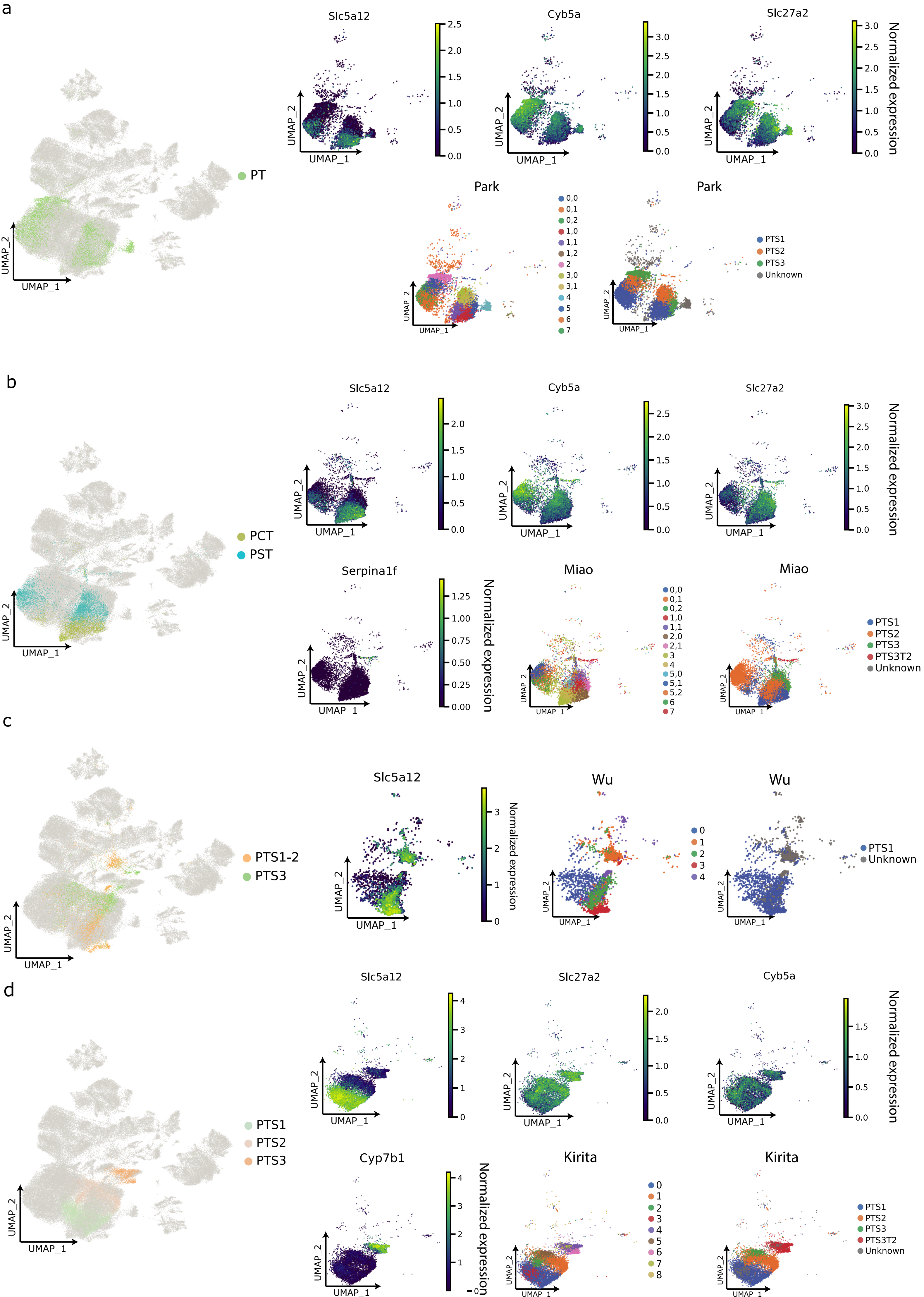

Figure S3. Integrated atlas allows further division of proximal tubule cells. (a-d) UMAP plots for Park18 (a), Wu19 (b), Miao21 (c) and Kirita20 (d) highlighting *PT*, *PTS1*, *PTS2* and/or *PTS3* (top left), their marker expression (top right; PTS1: *Slc5a12;* PTS2: *Cyb5a;* PTS3: *Slc27a2,* PTS3T2: *Cyp7b1*) and the unsupervised clusters (bottom left). Clusters are renamed to *PTS1*, *PTS2*, *PTS3* or *PTS3T2* according to the marker expression overlay (bottom right). If the given marker has no expression on the dataset, the corresponding UMAP plot is omitted. *PT*: Proximal Tubule, *PTS1*: Proximal Tubule Segment 1, *PTS2*: Proximal Tubule Segment 2, *PTS3*: Proximal Tubule Segment 3, *PTS3T2*: Proximal Tubule Segment 3 Type 2.

Supplementary Figure 4

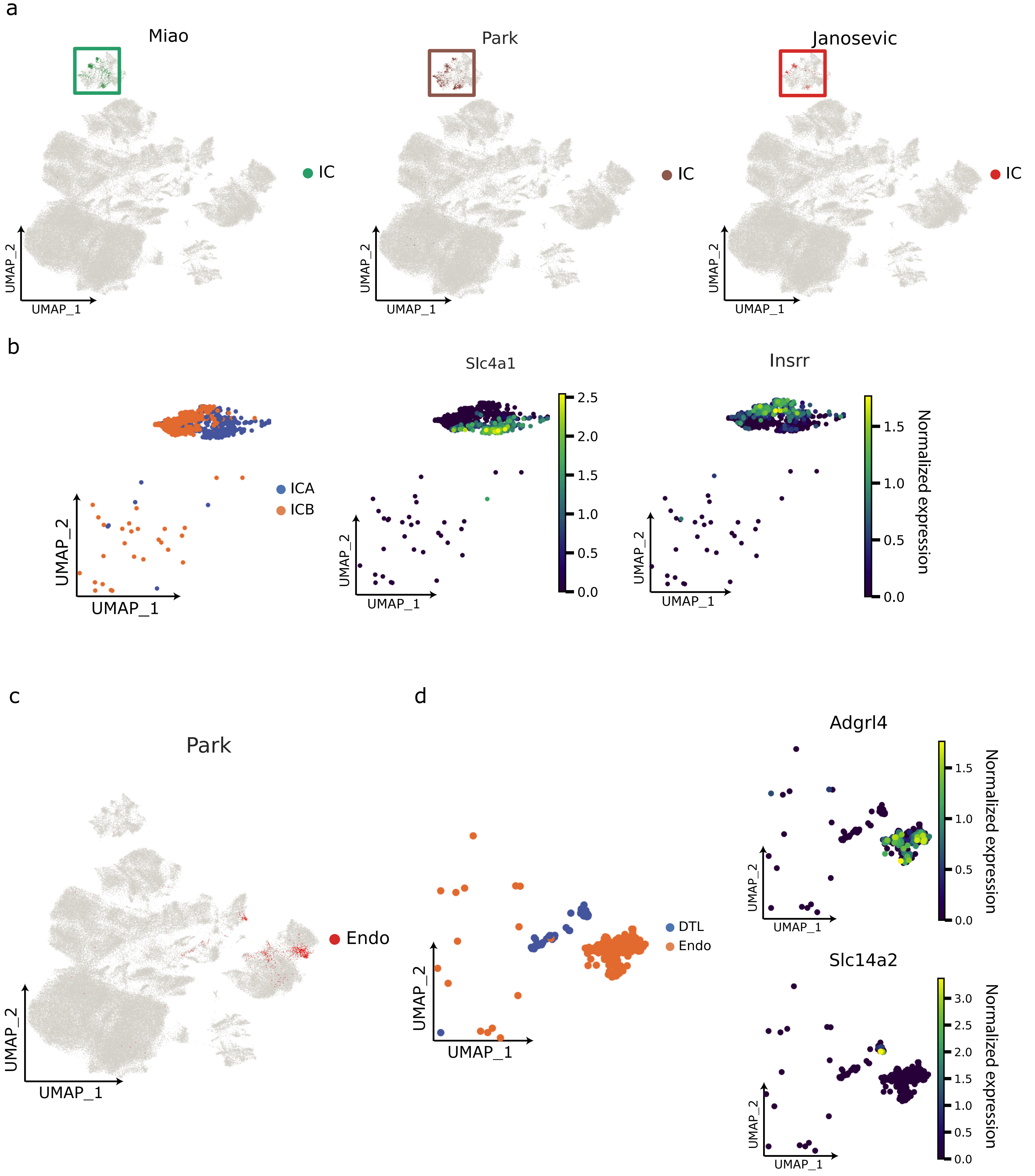

Figure S4. Further division of collecting duct intercalated cells and endothelial cells. (a-b) UMAP plots coloured by IC_{Miao21} IC_{Park18} and $IC_{Janosevic21}$ (a) and $Endothelial_{Park18}$ cells (b). **(c)** UMAP plot coloured by the Endothelial cluster **(d)** UMAP plots coloured by renamed cluster (left) and marker expression (right) of *IC* (top) or *Endothelial* cells (bottom). Clusters are renamed to *ICA*, *ICB*, *Endothelial* or *DTL* according to the marker expression overlay (*ICA*: *Slc4a1*; *ICB*: *Insrr*; *Endo*: *Adgrl4*, DTL: *Slc14a2*).

Supplementary Figure 5

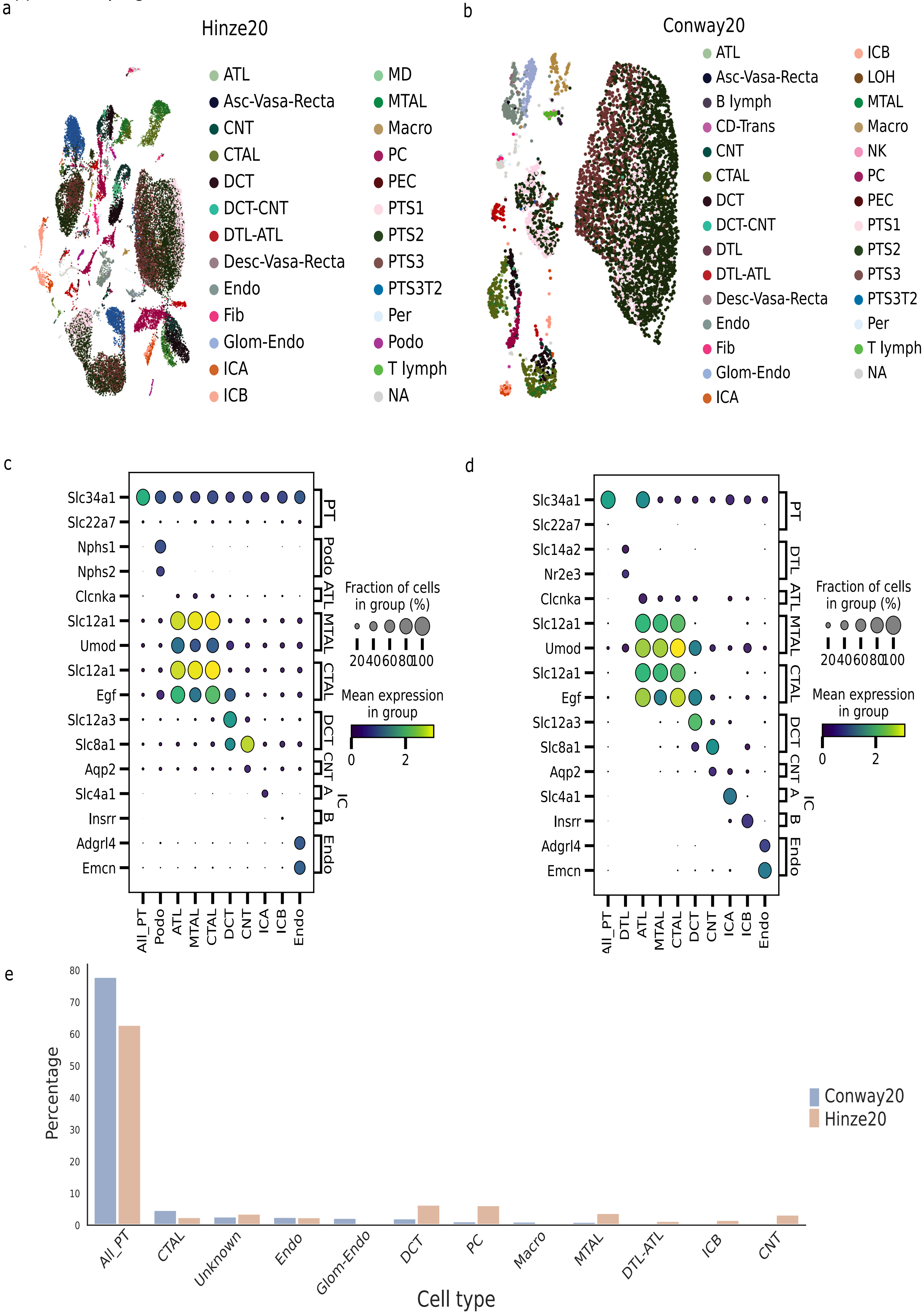

Figure S5. Cell type prediction per dataset. (a-b) The final learned hierarchy (classification) tree was used to individually predict the cell types of the unannotated cells in the used datasets in the atlas. **(c-d)** Dotplot of predefined set of standard markers in every predicted population per dataset. *IC*: Intercalated Cell, *ICA*: Intercalated Cell Type A, *ICB*: Intercalated Cell Type B, *Endo*: Endothelial Cell, *Fib*: Fibroblast, *Macro*: Macrophage, *B lymph*: B lymphocyte, *Stroma*: Stroma cell, *NK*: Natural Killer*, T lymph*: T lymphocyte, *PT*: Proximal Tubule, *PTS1*: Proximal Tubule Segment 1, *PTS2*: Proximal Tubule Segment 2, *PTS3*: Proximal Tubule Segment 3, *PC*: Principal Cell, *PEC*: Parietal Epithelial Cell, *Per*: Pericyte, *DCT*: Distal Convoluted Tubule, *ATL*: Ascending Thin Limb of Henle, *MD*: Macula Densa, *LOH*: Loop of Henle, *CTAL*: Thick Ascending Limb of Henle in Cortex, *MTAL*: Thick Ascending Limb of Henle in Medulla, *CNT*: Connecting Tubule, *Podo*: Podocyte, *DTL*: Descending Thin Limb of Henle, *MC*: Mesangial Cell, *Neutro*: Neutrophil, *Asc-Vas-Recta (Asc VR)*: Ascending Vasa Recta, *Desc-Vas-Recta (Desc VR)*: Descending Vasa Recta, *Glom Endo*: Glomeruli Endothelial.

Supplementary Figure 6

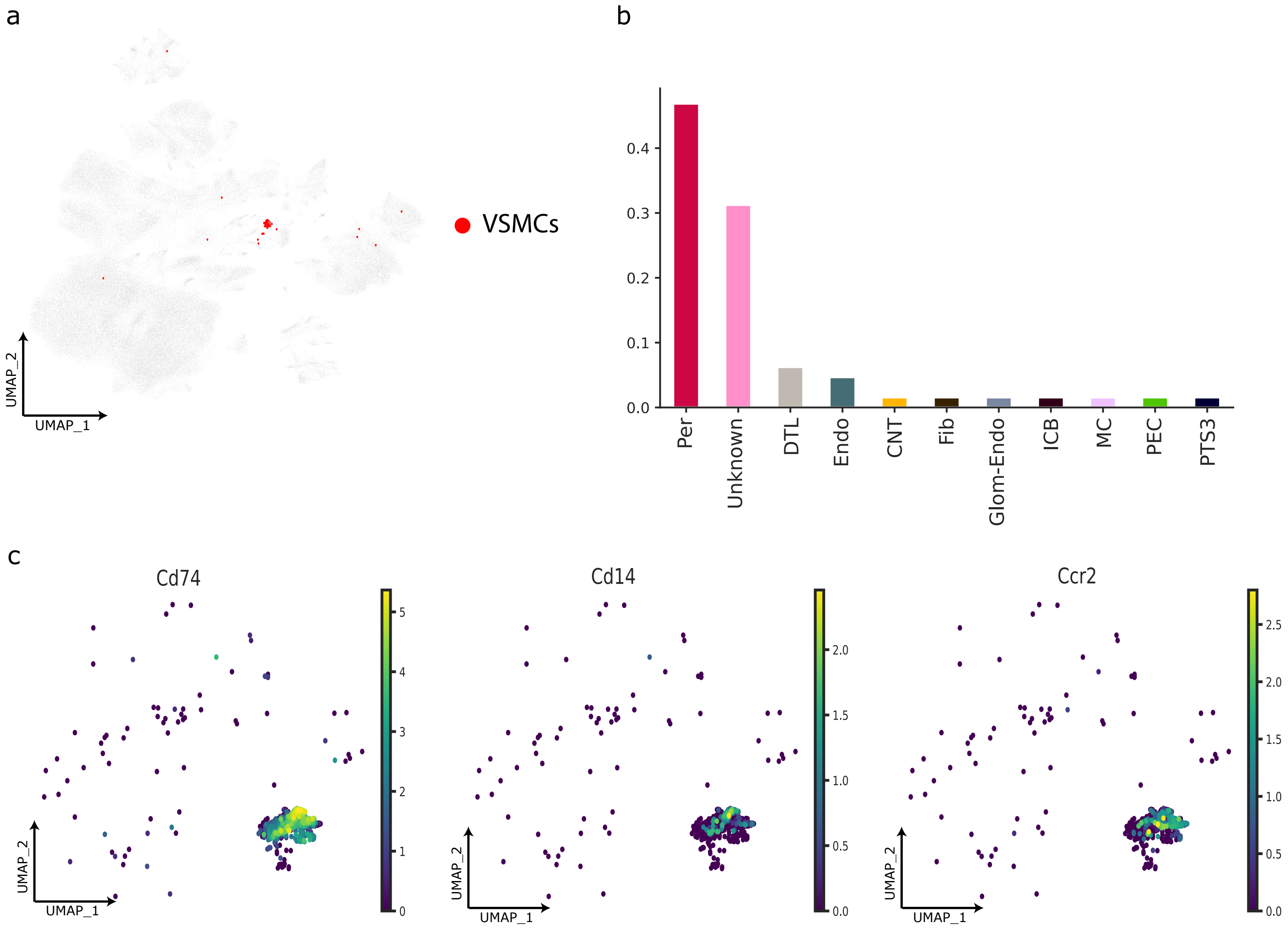

Figure S6. Identification of additional subpopulations. (a) UMAP plot coloured by VSMCs, defined as cells expressing both *Acta2* and *Myh11*. **(b)** Bar plot with the % of cell type labels present in the 689 detected VSMCs. **(c)** UMAP plots of Macrophages coloured by the log-normalized expression of Macrophage marker (*Cd74*) and Infiltrating monocyte markers (*Cd14* and *Ccr2*).

Supplementary Figure 7

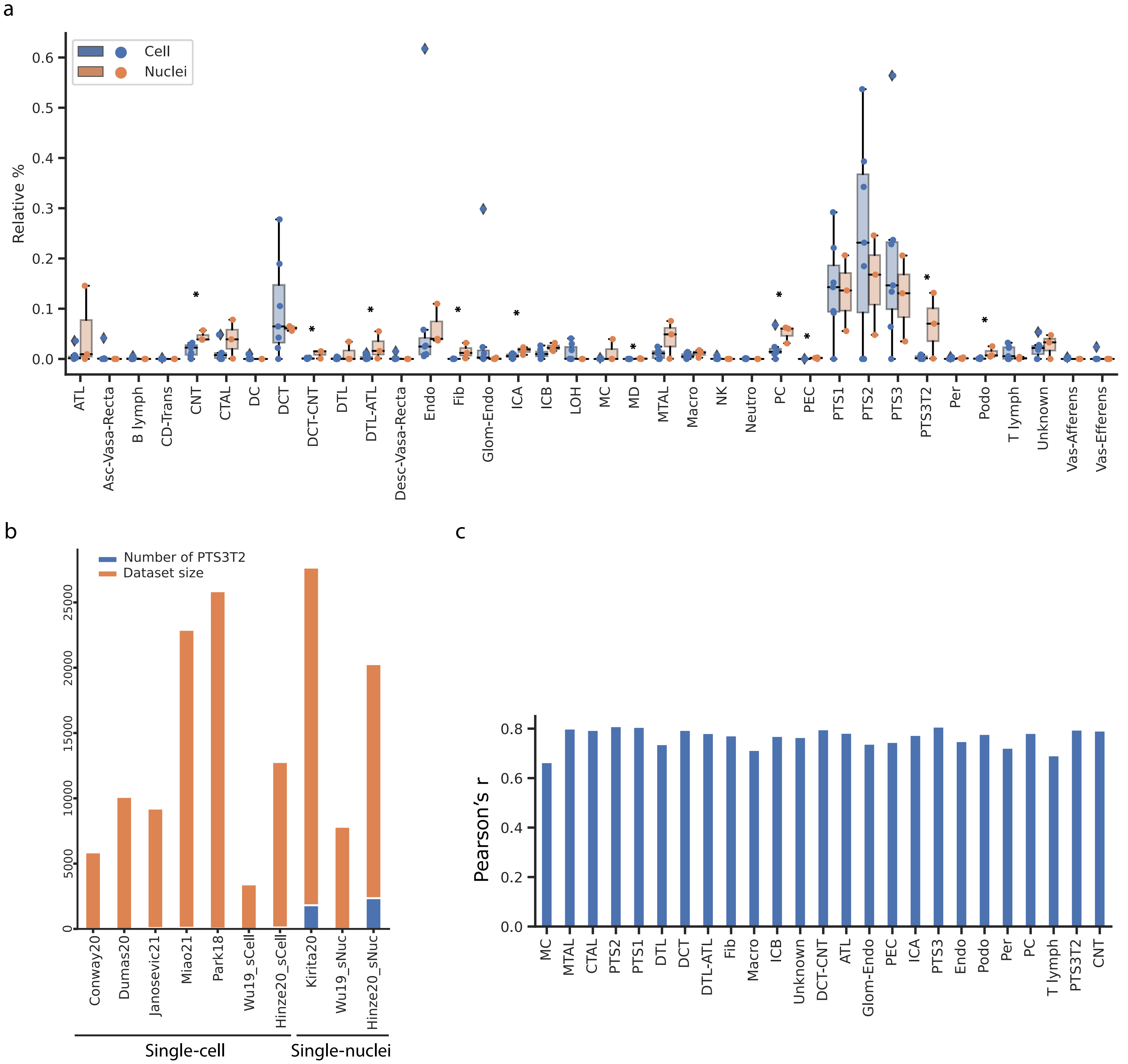

Figure S7. Single-cell and single-nuclei contribution to the MKA. (a) Boxplot showing the relative contribution (compared to the total amount of cells and or nucleus present in each dataset) of single-cells and single-nucleus to each of the cell types present in the MKA. Each dot represents a dataset in the MKA. Blue dots correspond to single-cell datasets whereas orange dots correspond to single-nuclei datasets. *: p-value < 0.05, two-sided T-test. **(b)** Stacked barplot showing the total amount of PTS3T2 cells or nuclei detected (blue) per dataset, with their respective total size (orange). (c) Barplot showing Pearson's coefficient of correlation between the batch-corrected expression profile of single-cells and single-nucleus for a given cell type.

Supplementary Figure 8

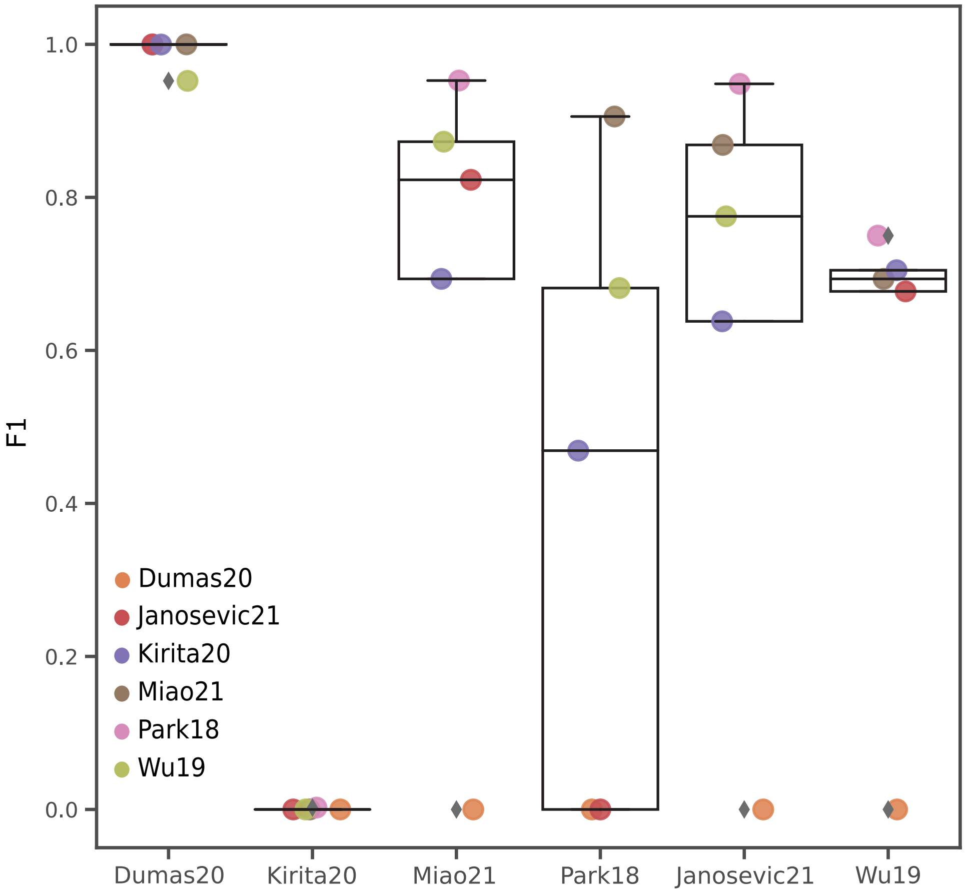

Figure S8. Accuracy of predictions using single-dataset references. Boxplots of median F1 scores (x-axis) computed over five single-dataset references for all annotated datasets in MKA (y-axis). Colours indicate the single dataset used as reference in Azimuth's label transfer workflow.

Supplementary Figure 9

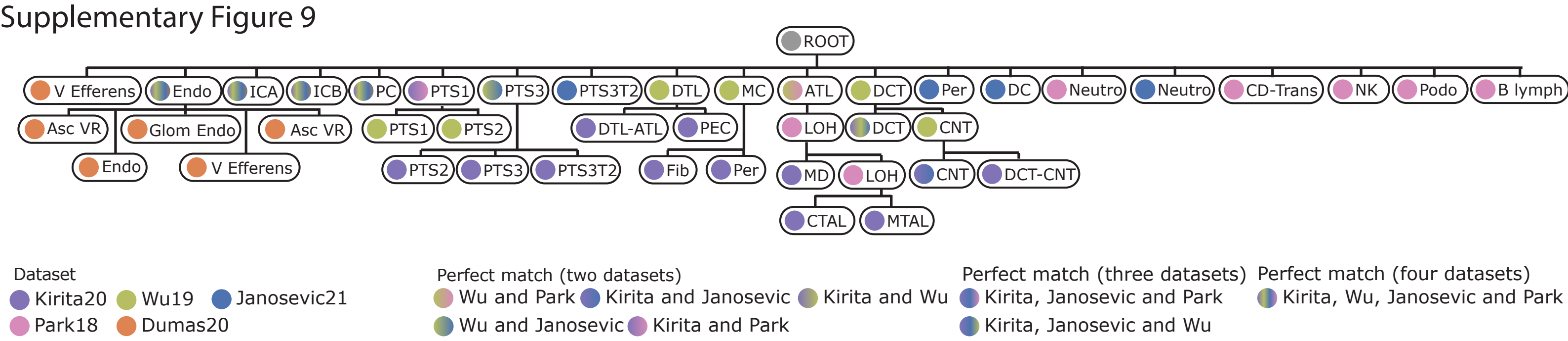

Figure S9. MKA*'s learned classification tree. Tree built by training a k-Nearest Neighbor (kNN) classifier on five of the six annotated datasets (Miao21 dataset was excluded, MKA*). The colour(s) of the tree nodes represent the agreement with the supporting dataset(s)

Supplementary Figure 10

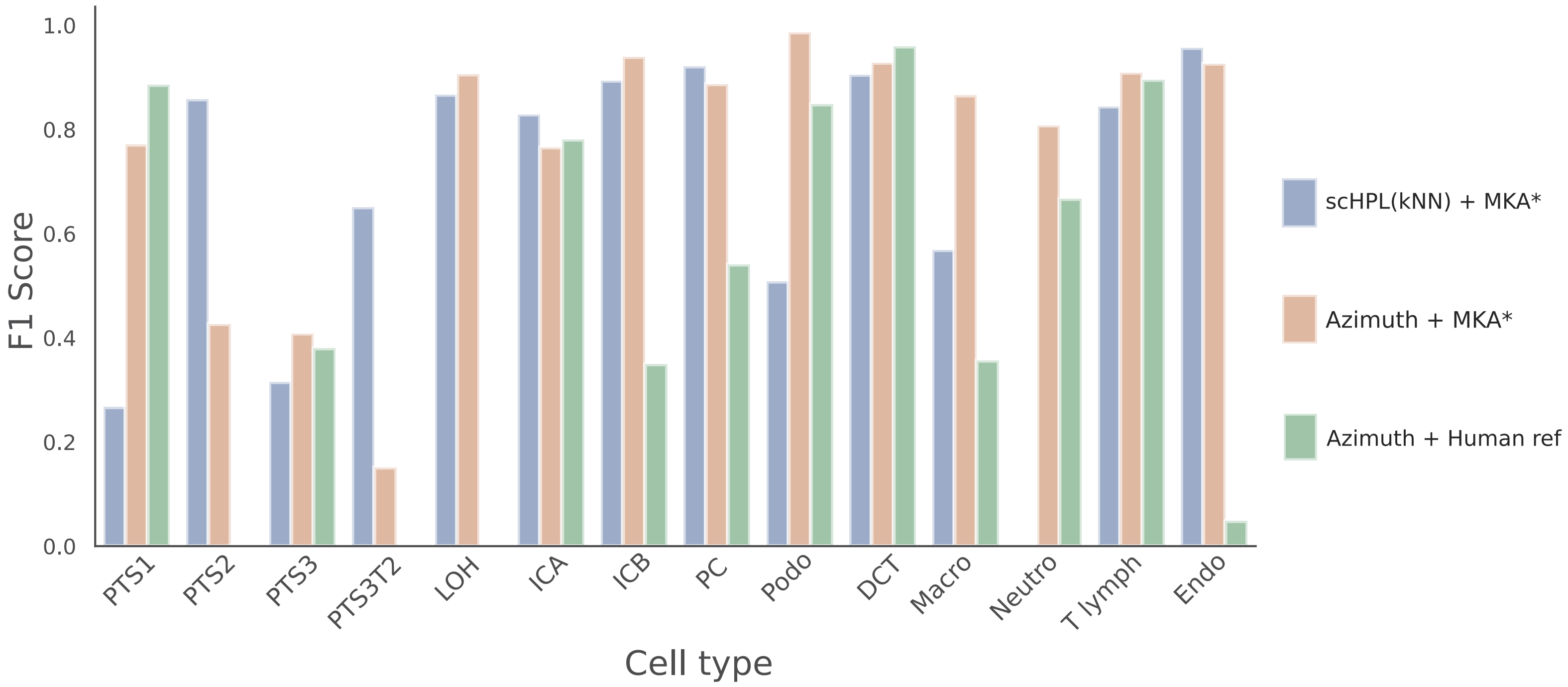

Figure S10. F1 scores per cell type and evaluation experiment. For each cell type, the F1 score is plotted for the different Miao21 classification tasks. Namely, scHPL trained with our partial reference mouse atlas, Azimuth trained with a human reference and Azimuth trained with our partial reference mouse atlas.

Supplementary Figure 11

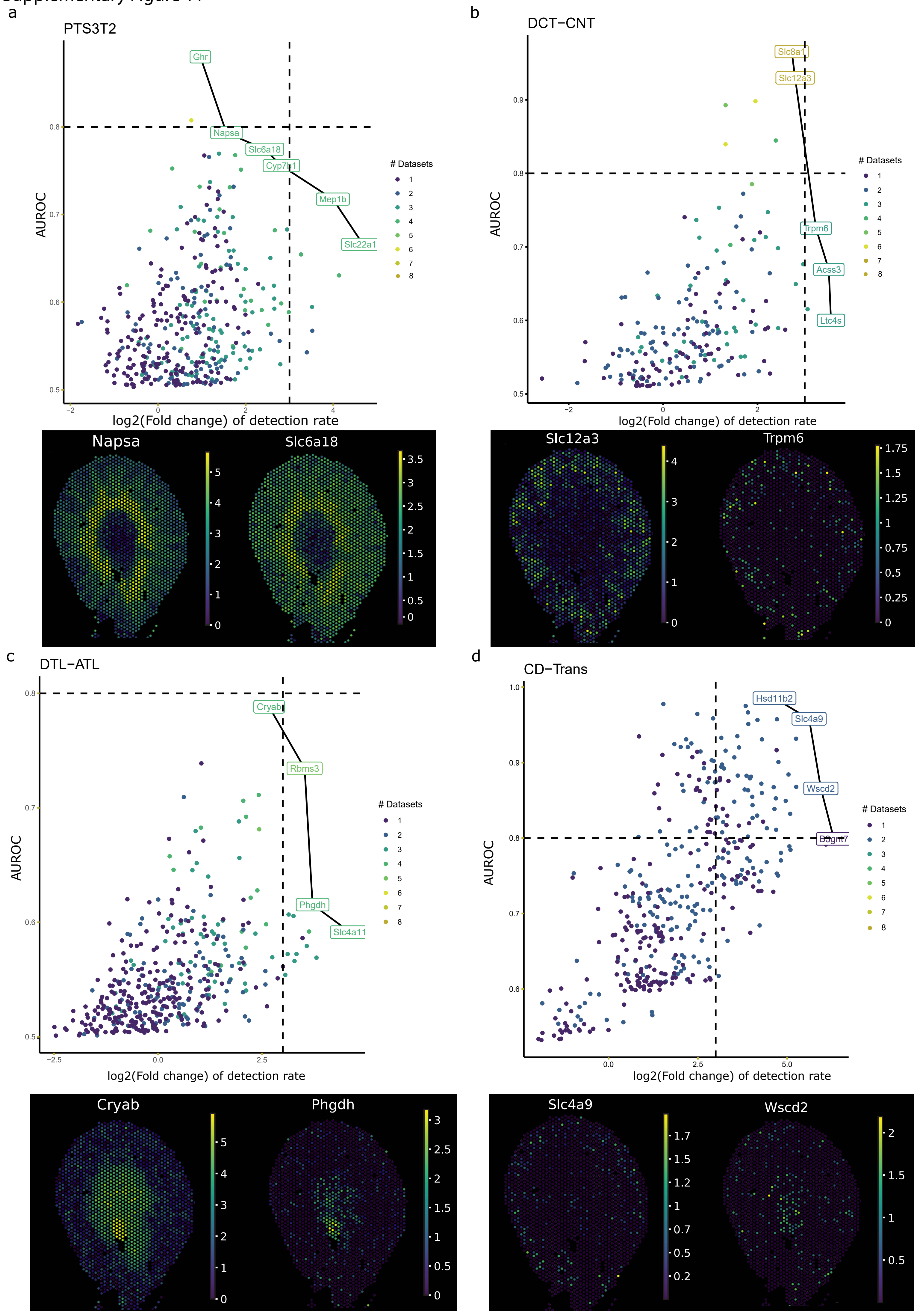

Figure S11. Meta-markers of transitional cell types and rare populations. (a-d) Each dot represents a significant meta-marker (FDR < 0.05). Top markers with respect to detection rate (expressed as log2 Fold change) and precision (area under the receiver-operator curve; AUROC) for poorly described cell types are highlighted with a connected boundary line. Bottom panels show the log-normalized expression of two of these meta-markers in a healthy spatial transcriptomics kidney slide for every cell type. *PTS3T2*: Proximal Tubule Segment 3 Type 2, *DCT*: Distal Convoluted Tubule, *ATL*: Ascending Thin Limb of Henle, *CNT*: Connecting Tubule , *DTL*: Descending Thin Limb of Henle; *CD-Trans*: collecting duct transitional cell population

Supplementary Figure 12

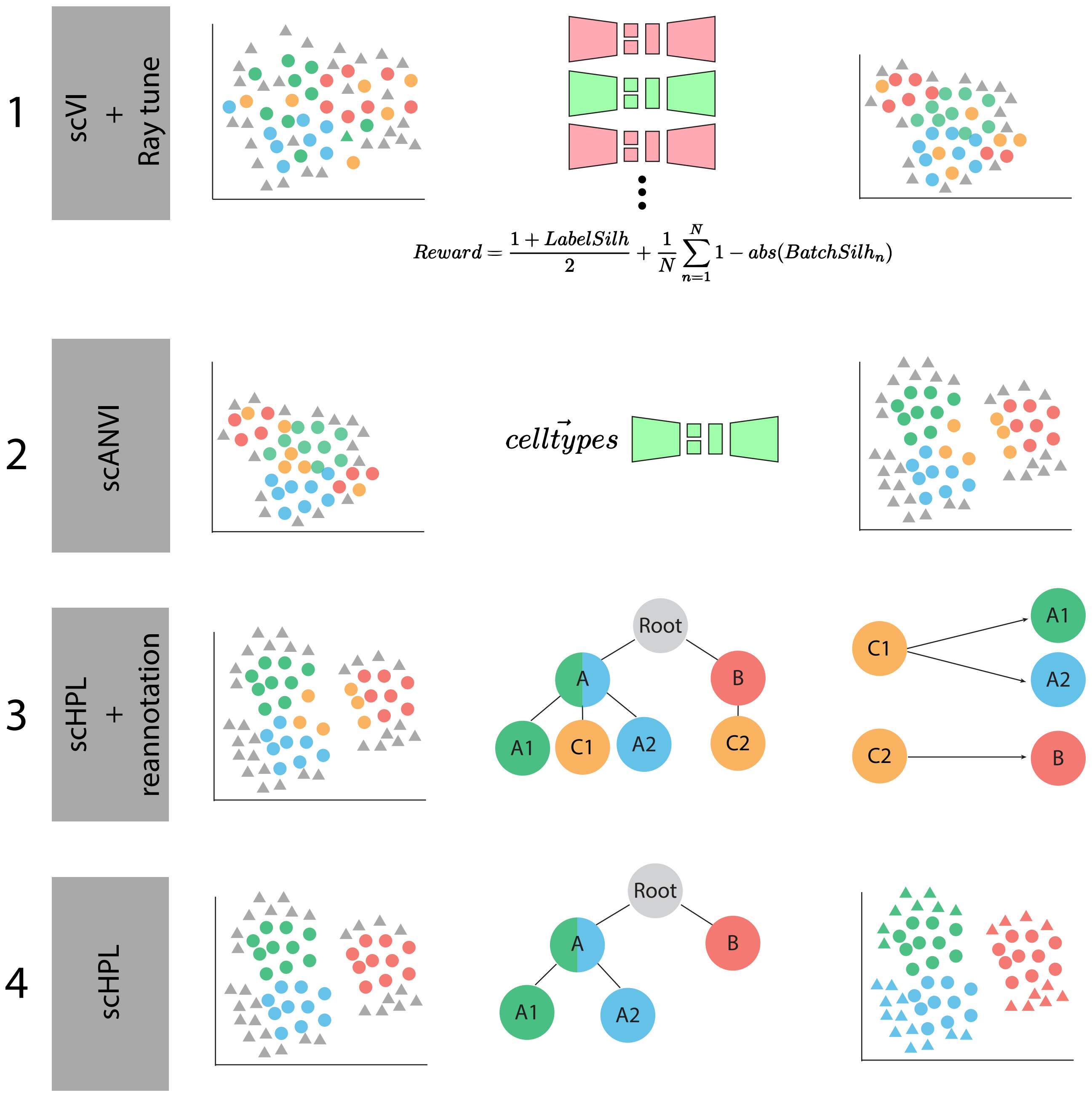

Figure S12. MKA pipeline. Colours represent different hypothetical cell type annotations from two independent studies (A, B, A1, A2) and two mislabelled cell types (C1 and C2). Shapes depict originally annotated (circle) or unannotated (triangles) cells and/or nuclei. Funnel illustrations represent a Variational Autoencoder architecture. Red funnels have suboptimal hyperparameter combinations according to the reward function. The green funnel indicates the VAE architecture and hyperparameter combination that yielded the best batch mixing and cell type separation in the latent space. The vector cell types is a 1-dimensional array with cell type labels.
